# Supplementary material for: Diagnostic utility of DNA methylation analysis in genetically unsolved pediatric epilepsies and CHD2 episignature refinement
Source: Nat Commun. 2024 Aug 6;15:6524. doi: 10.1038/s41467-024-50159-6 (PMC11303402; doi:10.1038/s41467-024-50159-6)
Supplement: Supplementary file 1 — Supplementary Information [file 41467_2024_50159_MOESM1_ESM.pdf]

Supplementary Information

"Diagnostic Utility of Genome-wide DNA Methylation Analysis in Genetically Unsolved Developmental and Epileptic Encephalopathies and Refinement of a CHD2 Episignature"

by LaFlamme et al.

Supplementary Phenotype data

Epivariants:

chr13 hypermethylation and X;13 translocation: Individual T25808

Individual T25808 had hypermethylation along the chr13q and a corresponding translocation 46,XX,t(X;13)(q28;q14.2). This girl died at 7 months with the epilepsy syndrome of Epilepsy of Infancy with Migrating Focal seizures. Her development slowed from 6 weeks of age with loss of head and trunk control and decreased interaction. Explosive onset of focal seizures occurred at at 5.5 months, resulting in a 7-week admission until she died. EEG showed multifocal and generalized epileptiform activity; seizure recordings showed ictal migration between hemispheres. MRI was normal at 6 months. T25808 did not have any reports of retinoblastoma.

The clinical spectrum of chromosome 13 copy number variation underscores its dosage sensitivity. For instance, individuals with Trisomy 13 typically have multiple congenital anomalies, including heart defects, microphthalmia, polydactyly, cleft lip/palate, hypotonia, and severe intellectual disability[1]. Many individuals born with Trisomy 13 die in the first year of life. Individuals with partial monosomy 13 have been reported to have multiple congenital anomalies, craniofacial differences, intellectual disability, and retinoblastoma. While partial monosomy 13 is more like Individual T25808, her chromosome 13 hypermethylation and any subsequent deactivation is dependent on X chromosome inactivation, and, therefore, would not be uniform throughout all cells. Interestingly, another individual with a balanced X;13 translocation of a similar region to our case [46,XX,t(X;13)(q28;q14.1)] had failure to thrive, developmental delay, and unilateral retinoblastoma, without seizures[2].

***CSNK1E* hypermethylation/repeat expansion:**

Family 1: Individual T1440

Individual T1440 had harbor hypermethylation of the 5'UTR and intron 1 of *CSNK1E* due to an underlying repeat expansion. T1440 is a male (with an unaffected dizygotic male twin). He presented with a brief <1 minute tonic-clonic seizure at 5 years. He had 5-15 second staring spells at 6 years that resolved with valproic acid. He had learning difficulties noted at 4 years, and developmental regression at 5 and 6 years with loss of understanding of his name or instructions, and his speech deteriorated to monosyllabic words. MRI showed a right frontal venous angioma and diffuse bilateral parietal parenchymal loss. EEG showed bilaterally synchronous slow spike-wave activity in about 50% of the sleep recording. Individual T1440's mother also has hypermethylation and repeat expansion in *CSNK1E;* she has no known epilepsy or neurodevelopmental symptoms.

Family 2: Individual 34167

Individual 34167 had hypermethylation of the 5'UTR and intron 1 of *CSNK1E* and is a female with moderate intellectual disability who had onset of epileptic spasms around 9 months. She had apparently normal development until seizure onset, at which time speech (babble) and motor milestones (sitting, rolling) were lost. Epileptic spasms occurred in clusters associated with sleep. They had flexor semiology, and initially responded well to ACTH. Recurrence of epileptic spasms and onset of tonic seizures occurred at 18 months. At the last evaluation (12 years old), seizure burden had been stable since the age of 3.5 years (4 clusters per month/1 per week). MRI was normal. EEG findings included generalized spike wave, poly spike and slow wave, multi-focal discharges, and abnormal background prior to initiation of ketogenic diet, which resulted in a reported 90% improvement of the EEG.

Family 3: Individual UDN821227

Individual UDN821227 was found to harbor hypermethylation of the 5'UTR and intron 1 of *CSNK1E* due to an underlying repeat expansion. UDN821227 is a male with seizure onset around

3-4 years leading to developmental regression. Epilepsy includes drug-resistant seizures (tonic-clonic, myoclonic, atonic, and absence) with continuous spike and slow waves during slow sleep on EEG. He is described as having mild intellectual disability, gastrostomy tube placement at 3-years-old, poor speech, failure to thrive, abnormality of the uvula, abnormal epiglottis morphology, asthma, sleep apnea, restless sleep, history of gastroesophageal reflux, and recurrent infections. He has a diagnosis of Lennox-Gastaut syndrome.

Individual UDN821227's mother, who also has hypermethylation and a repeat expansion in *CSNK1E*, has no seizure history but has neurodevelopmental, cognitive, and sleep findings. She has a history of learning difficulties, including dyslexia, requiring placement in a special needs classroom from 4th grade onward. She reports speech difficulties, including slurred words and finding the right words. She also reports significant difficulties with sleep, including falling asleep and nighttime wakefulness that impact quality of life and daily functioning. MRI brain is reported as normal.

***DIP2B* hypermethylation/repeat expansion:** Individual 8245

Individual 8245 had hypermethylation of the promoter region and exon 1 of *DIP2B*. He is a male of normal intellect with short stature (Patient 8245[3]). He had Epilepsy with myoclonic-altonic seizures (EMATs), preceding febrile seizures, generalized tonic-clonic seizures (GTCS), atonic and absence seizures, with seizures settling at 13 years. Hypermethylation was also present in his unaffected mother.

Individuals with hypermethylation and CGG-repeat expansion in the *DIP2B* gene have widely variable features including, developmental delay, learning disabilities, seizures, craniofacial differences, and recurrent infections[4]. Of note, apparently healthy or unaffected individuals have also been found to have hypermethylation of this region with a population frequency of ~0.1%. Given the relatively high population frequency and inheritance, hypermethylation as a cause for this individual's phenotype is unclear.

***BCLAF3* hypermethylation/repeat expansion:** Individual 33109

Individual 33109 was found to harbor hypermethylation in exon 1 of *BLCAF3* due to an underlying repeat expansion. Individual 33109 is a male with focal developmental and epileptic encephalopathy, moderate intellectual disability, and autism spectrum disorder. He had normal development until seizure onset with tonic-clonic seizures at 3 years when development slowed. He had developmental regression at 4 years. He had drug-resistant epilepsy, including convulsive status epilepticus (3 years 10 months), focal tonic seizures and focal clonic seizures (4 years 2 months), atypical absence seizures (4 years), atonic seizures (5 years 8 months), and focal impaired awareness seizure (7 years). EEG showed a slow background with multifocal discharges.

In a previous sequencing analysis, an inherited *SCN1A* variant (NM_001165963.4:c.4096G>A, NP_001159435.1:p.Val1366Ile) was detected in 33109, his affected brother, and his unaffected mother. His 7-year-old brother had drug-responsive tonic-clonic seizures between 3 and 5 years of age with a normal EEG and MRI. He has normal cognition with autistic features. As the proband's mother is unaffected, the proband and his brother's phenotype are not consistent with *SCN1A*, and the variant is present in 5 individuals in gnomAD, it is thought that the *SCN1A* variant alone is not the cause of 33109's phenotype.

***STX1B* hypermethylation/deletion**: Individual 7067 and family

This family with Genetic Epilepsy with Febrile Seizures plus (GEFS+) had 7 affected individuals, with 5 undergoing molecular testing; there were another 7 more distant individuals with an unconfirmed history of seizures (Pedigree in Figure Supplementary Figure 12A). The proband (7067) is a male with Epilepsy with Myoclonic-Atonic Seizures (EMAtS) that evolved to Lennox-Gastaut syndrome in the setting of moderate intellectual disability, autism spectrum disorder and obstructive sleep apnea. At 14 months, he presented with GTCS and myoclonic seizures.

Convulsive status epilepticus occurred once at 18 months. Myoclonic-atonic, absence and tonic seizures began at 2 years and non-convulsive status epilepticus at 12 years. Development was normal prior to seizure onset, when he became more withdrawn. Seizure exacerbation at 4 years was associated with regression in writing his name. At 7 years, his EEG showed generalized polyspike-wave activity associated with myoclonic seizures. At 38 years, video-EEG monitoring captured slow spike-wave increasing in sleep, generalized paroxysmal fast activity, frequent tonic seizures with prominent apnea, atonic and clonic seizures. Neuroimaging (MRI and CT-brain) was normal. Dysmorphic features included lumbar lordosis, clinodactyly, large flat feet with prominent toes, and a large capillary haemangioma over the lumbar spine.

His sister (7068) had EMAtS and mild intellectual disability. She had GTCS with fever at 6 weeks. Later she had afebrile GTCS at 28 months, then developed myoclonic-atonic and absence seizures at 4 years. At 10 years, she developed focal impaired awareness seizures and non-convulsive status epilepticus. Development was always delayed and regression occurred at 8 years old on carbamazepine. An EEG at 12 years showed background slowing and generalized epileptiform activity. CT-brain was normal. She had myasthenia gravis and underwent thymectomy at 14 years.

Their father (7066), who died at >60 years, had epilepsy following a motor vehicle accident at 30 years. He had a possible febrile seizure at 18 months and, at 32 years, three generalized tonic-clonic seizures in sleep. EEG showed right anterior quadrant slowing, without epileptiform activity. MRI showed right frontal focal atrophy following the contusion and a large cisterna magna.

Their mother (7065) had Febrile Seizures Plus (FS+) with 10 febrile seizures from 6 months to 7 years of age, and afebrile GTCS from 11 years to 29 years. EEG showed left temporal slowing and CT brain was normal.

The maternal grandmother (7215) had temporal lobe epilepsy (TLE) with focal aware and focal to bilateral tonic-clonic seizures from 15.5 years. EEG showed left temporal slowing and MRI was normal.

The proband (7067), affected sister (7068), affected mother (7065), and affected maternal grandmother (7215) all exhibited outlier hypermethylation of the promoter and TSS of *STX1B* (Figure 3). This hypermethylation was found to be associated with an underlying heterozygous deletion encompassing the *STX1B* promoter, TSS, exon 1, and part of intron 1 [chr16:31,009,063-31,010,847x1 (GRCh38/hg38)], which segregated with the non-acquired epilepsy in the family. This family had three *CACNA1H* variants identified but no variant segregated perfectly through the family (Family A[5], Family B[6], Family A[7]).

Episignatures:

ANKRD11 **(KBGS_MRD23):** Individuals T23952 and 9714

Individual T23952 had a *de novo* pathogenic stop gain variant in *ANKRD11* (NM_001256183.2:c.2512C>T, NP_001243112.1:p.Arg838*). T23952 had developmental regression at 5 months with loss of eye contact. At 7 months, eyelid myoclonias were observed, followed by epileptic spasms, with seizure offset at 9 months. Facial features were characteristic of *ANKRD11* facies, including a triangular face, bulbous nose, thin upper lip, featureless philtrum, broad bushy eyebrows, large prominent ears, and thin upper lip (Figure S16). She was of borderline intellect and had anxiety.

Individual 9714 had a *de novo* pathogenic stop gain variant in *ANKRD11* (NM_001256183.2:c.6871G>T, NP_001243112.1:p.Glu2291*). She is a female who had neonatal onset of tonic-clonic seizures, which stopped at 18 years. Functional neurological seizures began at 27 years. She had developmental delay, and at 7 years she was of low average intellect.

Individuals with pathogenic *ANKRD11* variants (KBG syndrome) are reported to have unique facial dysmorphisms, skeletal anomalies, intellectual disabilities, behavioral issues, seizures, and brain malformations[8].

SETD1B **(IDDSELD):** Individual 36369

Individual 36369 was found to harbor a pathogenic stop gain variant in *SETD1B* (NM_001353345.2:c.4360C>T, NP_001340274.1:p.Arg1454*). 36369 is a male with absence with eyelid myoclonia seizure onset around 2 years old. At 10 years, he developed myoclonic seizures. Initially, he presented with hypotonia and developmental delay at 1 year of age. He has a moderate intellectual disability and is obese. EEG has shown generalized spike wave, polyspike and slow wave, and photosensitivity response (absence with eyelid myoclonia seizures triggered by eye closure). His father has temporal lobe epilepsy and was unavailable for genetic testing.

Individuals with *SETD1B*-related neurodevelopmental disorders are reported to have developmental delays (mainly affecting speech and language), intellectual disability, seizures (including absence with eyelid myoclonia seizures), autism spectrum disorder and autism-like behaviors, and additional behavioral concerns[9].

TET3 **(BEFAHRS):** Individual T22466

Individual T22466 was found to harbor a maternally inherited, likely pathogenic small indel in *TET3* (NM_001287491.2:c.5243dup, NP_001274420.1:p.Thr1749Hisfs*5). T22466 is a female with Developmental and Epileptic Encephalopathy and Spike-Wave Activation in Sleep (DEE-SWAS) and mild intellectual disability. She had mild gross motor and language delay in the first year of life. At 3 years 4 months she developed occasional focal to bilateral tonic-clonic seizures and focal impaired awareness seizures and her development progressed slowly. At 6 years she developed stuttering and language regression associated with frequent independent high

amplitude bi right and left temporal parietal discharges while awake (sleep not captured). She has macrocephaly (>98th centile), and MRI is normal. Her mother also has the *TET3* variant, a large head size (>90th centile), and learning difficulties; her maternal grandmother had epilepsy in childhood.

Individuals with *TET3*-related disorders have been reported to have variable phenotypes including global developmental delay, intellectual disability, dysmorphisms, and multiple congenital anomalies[10].

UBE2A **(MRXSN):** Individual 26720

Individual 26720 had a maternally inherited, likely pathogenic variant in *UBE2A* (NM_032590.5:c.376G>A, NP_115979.3:p.Ala126Thr). This adolescent had onset of GTCS at 28 months. At 6 years, he developed focal seizures and focal to bilateral tonic-clonic seizures. He had developmental delay without regression, and now has mild intellectual disability and ADHD. He had dysarthria and dysmorphic features including a short, upturned nose with a flattened nasal bridge, short well-defined philtrum with a tented upper everted lip, high arched eyebrows, full cheeks, two anterior left-sided ear tags, short digits, short broad feet and hypoplastic nails. EEG showed bilateral independent centrotemporal discharges. MRI at 2 years showed sub-cortical white matter signal change.

Individuals with *UBE2A*-related X-linked intellectual disability have been reported to have facial dysmorphisms, hirsutism, hypoplastic genitalia, short stature, hypotonia, seizures, severe intellectual disability[11].

SMS **(MRXSSR)**: individual 31067

Individual 31067 had a maternally inherited, likely pathogenic variant in SMS (NM_004595.5:c.328C>G, NP_004586.2:p.Arg110Gly). 31067 is a male with Developmental and Epileptic Encephalopathy and moderate intellectual disability. Absence seizures were diagnosed at 17 months but may have been occurring as early as 6 months. Other seizure types include frequent myoclonic seizures and rare generalized tonic-clonic seizures. EEG showed generalized spike-wave and polyspike-wave activity with background slowing. His development was delayed at 6 months and regression occurred at age 2.5 years. He had speech dyspraxia with nasal speech, hypotonic cerebral palsy, and an ataxic gait. Dysmorphic features included friable hair, moderately high arched palate, thin upper lip, fine eyebrows, low anterior hairline and long, thin fingers and toes. He has osteoporosis with a history of fractures. MRI brain was normal.

Individuals with *SMS*-related X-linked intellectual disability disorders (Snyder-Robinson Syndrome/Spermine Synthase Deficiency) have been reported to have hypotonia, developmental delays and moderate to and severe intellectual disability, speech abnormalities (nasal, dysarthric, coarse, or absent), facial dysmorphisms (prominent lower lip; high, narrow, or cleft palate), asthenic (long and narrow) build, osteoporosis leading to fractures, kyphoscoliosis, short stature, variable motor disability and gait abnormalities, and seizures[12].

***KDM2B* (KDM2B):** Individual 33769

Individual 33769 was found to harbor a paternally inherited variant in *KDM2B* (NM_032590.5:c.1046G>C, NP_115979.3:p.Arg349Pro). Individual 33769 displays macrocephaly and focal epilepsy, consistent with their diagnosis of *PTEN* (NM_000314.8:c.253+5G>A, *de novo*). The individual's father (33770) is unaffected, and, therefore, the paternally inherited *KDM2B* variant is unlikely to contribute to the individual's phenotype.

Episignature Negative with *CHD2* VUS: Individual ILGC_1081

Individual ILGC_1081 had normal development until 10 years of age. Seizure onset was at 6 years of age with focal semiology from the left temporal lobe. She was found to harbor a VUS in *CHD2* (NM_001271.4:c.5002C>T, NP_001262.3:p.His1668Tyr) that was not maternally inherited, but her father was unavailable for testing. Because ILGC_1081 was found to be negative for the *CHD2* episignature in conjunction with a lack of clinical evidence for *CHD2*-encephalopathy (i.e. absence of myoclonic or photosensitive seizures), the *CHD2* variant was determined to be likely benign. A maternally inherited pathogenic *CACNA1A* variant (NM_001127221.1:c.5559_5560delCA, NP_001120693.1:p.Tyr1853*) was also found. Her maternal side of the family has other affected members (half-sibling with febrile seizures, maternal aunt, and maternal grandmother with epilepsy, not genetically tested). Finally, her MRI was remarkable for mild vermian hypoplasia, which can be seen in calcium channel diagnoses. Thus, the pathogenic variant in *CACNA1A* was determined to be more consistent with her clinical phenotype. Her seizures have been intractable to multiple medications and placement of a responsive neurostimulation device.

Supplementary Methods

Cohort Subjects and Controls

Unaffected, presumably healthy controls without DEEs include 111 healthy controls obtained through the Parkinson's Progression Markers Initiative (PPMI)[13], institutionally available data for 335 community control individuals without cancer from the St. Jude Life (SJLIFE) study[14], and 29 unaffected parents or siblings of participants in our study cohort (Supplementary Data 1). Analytical controls include six individuals with known rare DMRs for validation of the computational outlier approach. These include two probands with Baratela-Scott syndrome (BSS) harboring *XYLT1* heterozygous hypermethylation on one allele and pathogenic variants on the other allele, one heterozygous *XYLT1* hypermethylation carrier mother of child with BSS, and three individuals with Fragile X syndrome harboring *FMR1* hypermethylation (two males, one female) obtained from the Coriell Institute for Medical Research (Table S1). Further analytical controls include 26 individuals with DEEs caused by pathogenic variants in genes with known episignatures to validate the episignature screening, including n=17 *CHD2*, n=1 *CHD2* VUS, n=1 *KDM5C*, n=1 *SETD1B*, n=1 *KMT2A*, n=1 *SMARCA2*, n=2 *SMC1A*, and n=2 CNVs (Table S4). Finally, we included 116 analytical controls comprised of individuals with DEEs who had previously identified pathogenic variants in genes without known episignatures. These 116 individuals better age match our unsolved DEE cohort and were used as further controls for comparison of rare DMRs and episignatures.

A short application for access to the PPMI dataset is required. This may be found by navigating to https://www.ppmi-info.org/ and choosing to “apply for data access.” Once granted, details about the sample collection and methylation array data acquisition are given as “genetic data” under “methylation profiling” in a PDF called <Project 140: Comprehensive Methylation Profiling Methods.pdf>. Sample distribution and other qualitative metadata for the cohort may be found in “study data” under “subject characteristics.”

Reproducibility of DNA Methylation Array Replicates for Outlier Analysis

For individuals with replicate array data (n=29 individuals, n=61 total samples), we checked for the reproducibility of autosomal DMRs across different batches for the same individual. Of the 29 individuals, 17 of the individuals had perfect concordance across replicates for the DMRs that were called by the algorithm; six individuals had DMRs called for one replicate that could be confirmed through manual inspection. The remaining 6 individuals had at least 1 DMR that was not reproducible across replicates. Of the total number of DMRs for the 61 replicate samples, 80% were reproduced across the replicates.

Targeted Enzymatic Methyl-Sequencing Library Preparation, Target Enrichment, and Sequencing

This approach uses two sets of enzymatic reactions to convert unmodified cytosines to uracil, allowing for the detection of methylated vs.unmethylated cytosines without the need for bisulfite conversion[15].

Approximately 200-300ng of peripheral blood-derived genomic DNA input for 9 samples (three positive controls (*XYLT1* and *FMR1* hypermethylation) and three DEE probands with DMRs-of-interest) were diluted to 52µL with 0.1X TE pH 8.0 in a 96 microTUBE-50 AFA fiber plate (PN 520168) and fragmented on the Covaris LE 220 with SonoLab 7.3 software (Covaris Inc.). A 120-second program with peak incident power (W) of 453.0, duty factor (%) of 15.2, and cycles per burst (cpb) of 1000 resulted in an average fragment size of 285 bp. Then, 50µL of each sample was transferred to a fresh plate to begin library construction. End repair, A-tailing, and the ligation of the 0.4µM EM-Seq adaptor (A5mCA5mCT5mCTTT5mC5mC%mCTA5mCA5mCGA5mCG5mCT5mCTT5mC5mCGAT5mC*T and [Phos]GAT5mCGGAAGAG5mCA5mCA5mCGT5mCTGAA5mCT5mC5mCAGT5mCA) were performed with NEBNext DNA Ultra II reagents according to the manufacturer's instructions

(PN 101977). Products were then purified by 1.18x bead clean up, using Twist Total Purification Beads (PN 101979) and eluted in 30µL water. About 28µL of each eluate was used as input for the 50µL oxidation reaction by TET2, proteinase K stop digestion, 1.8x bead clean-up, formamide denaturation, APOBEC deamination, and 1.0x bead purification described previously[15]. Then, 20µL of each deaminated library was used as input in an amplification reaction with 25µL of NEBNext Q5U Master Mix (#M0597) and 1µM (5µL) of primer from NEBs 96 Unique Dual Index Primer Pairs Plate (#E7166A) as follows: 30 sec at 98°C; cycling 10 times, 10 sec at 98°C, 30 sec at 62°C, and 60 sec at 65°C; with a final extension for 5 minutes and hold at 4°C.

A 0.9x bead purification was performed on each amplified library, and 20µL of each eluate was used as input for target enrichment with the Twist Biosciences Fixed Human Methylome Panel. The 8-plex hybridization captures were created with 200ng of each sample combined to create a 1.6ug pool. Then, 4µL of Twist's Fixed Methylome Panel (PN 105521), 8µL of Universal Blockers, 5µL of Blocker Solution (#100767), and 2µL of Methylation Enhancer (#103558) were added to each pool and then dried down using an Eppendorf Vacufuge Plus with V-AQ setting @ 45°C for approximately 1 hour to create a pre-hybridization solution. Twist's Fast Hybridization mix (PN 104182) was heated at 65°C for at least 10 minutes until all precipitates dissolved, and 20µL was added directly to each of the dried pools without allowing the reagent to cool. Then, 30µL of Hybridization Enhancer was added to the top of each pool, and these pools were transferred to a thermal cycler set to a 95°C hold. Once the lid was sealed, a 95°C hold for 5 minutes was initiated, and samples were subsequently held overnight at 60°C for a minimum of 16 hours. Then, 100µL of Streptavidin Binding Beads (#100984) were washed with 200µL RT Fast Binding Buffer, placed on a magnet for 1 minute, supernatant removed, for a total of 3 washes, a final 200µL FBB was added, and the beads were resuspended by vortexing. Each hybridization pool (8-plex) was added directly from the 60°C thermocycler to a tube of washed, resuspended Streptavidin Binding Beads and mixed at RT on a rotisserie axel for 30 minutes. Pools were then pulse spun and placed on a magnet for 1 minute. The supernatants were

removed and discarded. Tubes were removed from their magnets, and 200µL of preheated (62°C) Fast Wash Buffer 1 was added to each pool and pipette mixed. Pools were then incubated at 62°C for 5 minutes, pelleted on magnets for 1 minute, and the supernatant removed and discarded. Then, 200µL FWB1 addition and incubation were repeated, tubes were spun down, and bead-sample mixtures were transferred to fresh 1.5mL tubes before placing them on magnets. The remainder of the washes and final elution of enriched 8-plex libraries were performed according to the manufacturer's instructions (Twist Biosciences). Then, 30µL of each final enriched library was transferred to a clean tube for Illumina short-read sequencing.

All inputs and libraries were evaluated and quantified using D1000HS tape for TapeStation (Agilent) and Qubit™ 1X dsDNA HS (Invitrogen) assay kits. The average final library size was 370 bp. Sequencing was performed on the NovaSeq 6000 platform in paired-end mode with 150 bp per read. Paired-end reads (151 bp) for EM-Seq were collected from NovaSeq 6000 runsand then analyzed using bcl2fastq. The hg38_noAltHla_UCSC.fa reference file supplied through FTP by Twist Biosciences was used to create a reference genome in FASTA format. Samtools[16] and picard were used to create an index and ref sequence dictionary. The raw FASTQ data underwent quality control, processing and downstream analysis using the nf-core/methyseq bioinformatics pipeline. To extract the methylation calls using MethylDackel[17], we invoked the '—aligner bwameth' flag in the nf-core/methyseq pipeline. EM-Seq samples were trimmed using Trim Galore! (Krueger, Felix, 2015, v. 0.6.6) and cutadapt, and subsequently aligned using bwameth (default parameters), sambamba, and samtools. Duplicate reads were marked (picard) with an optical pixel distance of 2500 for a patterned S4 flow cell (Illumina). The covered_targets_Twist_Methylome_hg38_annotated_collapsed.bed file with TARGET and BAITS was converted to an interval list, and performance metrics were generated (fold-80 base penalty, HS library size, % duplicates, % off bait). MethylDackel was used to generate a methylation bias plot, likely variant sites were excluded (--maxVariantFrac 0.25), minimum depth of 10X (--minDepth 10) was set, and CpG calls were generated. Additional filters were added to

generate global methylation cytosine reports, including CHH and CHG sites. Mapping efficiency, mapped reads (samtools), and the global average CpG % methylation and non-CpG conversion ratio (%) for each sample were generated from the cytosine report (methyldackel).

Exome and Genome Sequencing

Variants for individual samples were called from ES and GS following the GATK best practices workflow (including VQSR)[18]. Minimum genotype quality (≥20), minimum coverage depth (≥7), and minimum variant allele frequency (≥20%) thresholds were applied with Bcftools[16]. Variants were annotated with population frequency data (gnomAD[19], ExAC[20], ESP6500[21], and 1000 Genomes[22]), in silico scores (dbNSFP[23]), and potential clinical relevance (ClinVar[24] and InterVar[25]) via ANNOVAR[24] and InterVar[25]) via ANNOVAR[26]. Additional in silico scores (LOFTEE[27], CADD[28], and SpliceAI[27]) were applied using VEP[28].

RNA-Sequencing and Gene Expression Analysis

RNA was extracted from flash-frozen cell pellets obtained from cultured fibroblasts using the Quick-RNA Miniprep Kit (Zymo Research). RNA was quantified using the Quant-iT RiboGreen RNA assay (ThermoFisher), and quality was checked by the 2100 Bioanalyzer RNA 6000 Nano assay (Agilent) or 4200 TapeStation High Sensitivity RNA ScreenTape assay (Agilent) before library generation. Libraries were prepared from total RNA with the TruSeq Stranded mRNA Library Prep Kit according to the manufacturer's instructions (Illumina PN 20020595). Libraries were analyzed for insert size distribution using the 2100 BioAnalyzer High Sensitivity kit (Agilent), 4200 TapeStation D1000 ScreenTape assay (Agilent), or 5300 Fragment Analyzer NGS fragment kit (Agilent). Libraries were quantified using the Quant-iT PicoGreen ds DNA assay (ThermoFisher) or by low-pass sequencing with a MiSeq nano kit (Illumina). Paired-end 100-cycle sequencing was performed on a NovaSeq 6000 (Illumina). The raw FASTQ data underwent processing and analysis utilizing the nf-core/rnaseq bioinformatics pipeline[29].

Whole-Genome Bisulfite Sequencing

The EZ-96 DNA Methylation-Gold MagPrep (Zymo Research) was used for bisulfite conversion. Libraries were prepared from converted DNA using the xGen Methylation-Sequencing DNA Library Preparation Kit (Integrated DNA Technologies). Libraries were analyzed for insert size distribution using the 2100 BioAnalyzer High Sensitivity kit (Agilent), 4200 TapeStation D1000 ScreenTape assay (Agilent), or 5300 Fragment Analyzer NGS fragment kit (Agilent). Libraries were quantified using the Quant-iT PicoGreen ds DNA assay (ThermoFisher) or by low-pass sequencing with a MiSeq nano kit (Illumina). Paired-end 150-cycle sequencing was performed on a NovaSeq 6000 (Illumina).

Functional Annotation and Enrichment Analysis of CHD2 Episignature Probes and DMRs

An in-house Perl script (https://github.com/MeffordLab/2024_GenomeWideMethylationPaper/blob/main/generate_random_genomic_ranges.pl) was used to systematically generate random genomic ranges (simulated genomic regions) for comparison in the enrichment analysis simulating background. The script, featuring a function named generate_genomic_ranges, allows for user-defined parameters such as the number of genomic ranges, minimum and maximum lengths, and a specified set of chromosomes. Chromosome information was extracted from a locally stored file containing the lengths of each chromosome. The script then randomly assigned start and end positions within the specified length constraints for each genomic range, associating them with randomly selected chromosomes. Subsequently, the resulting genomic ranges were sorted based on chromosome names and start positions. Finally, the sorted genomic ranges were exported to a BED file in tab-delimited format, facilitating downstream annotation for comprehensive analysis. To ensure robustness, we generated three distinct sets of simulated genomic regions (Replicate-1,

Replicate-2, and Replicate-3, Table S9), each comprising the same total number of regions as CHD2-Probes/DMRs (n=4767, Table S8) and of varying, comparable length (50bp-3,100bp), enabling meaningful comparisons in subsequent analyses.

For annotation purposes, we utilized the GREEN-DB[30] collection, encompassing various regulatory elements (bivalent regions, enhancers, and promoters sourced from ENCODE, FANTOM5, DiseaseEnhancers, BENGI, DECRES, etc.), transcription factor binding site (TFBS from UCSC genome browser tracks), and DNase peaks (from UCSC genome browser tracks). Direct links to these databases are now provided in https://github.com/MeffordLab/2024_GenomeWideMethylationPaper/blob/main/selected_GreenVarDB_links.sh. These annotations were applied to both the CHD2 Episignature Probes with Array and WGBS DMRs (Table S8) and the simulated replicate genomic regions (Table S9) using bedtools '-intersect' option with at least 90% overlap.

To assess the enrichment of features (Various regulatory elements, TFBS, and DNase sites) in the CHD2-Probes/DMRs, we computed the enrichment ratio (Table S10) with respect to the simulated genomic regions. This ratio was defined as the proportion of features in real regions divided by the total number of real regions, normalized by the proportion of features in simulated regions divided by the total number of simulated regions. Importantly, this method accounts for comparability in size and other relevant characteristics of genomic regions in both datasets. To validate the observed enrichment, two-sided Fisher's Exact tests (Table S10) were performed using the R statistical environment, ensuring the statistical robustness of the comparative analyses.

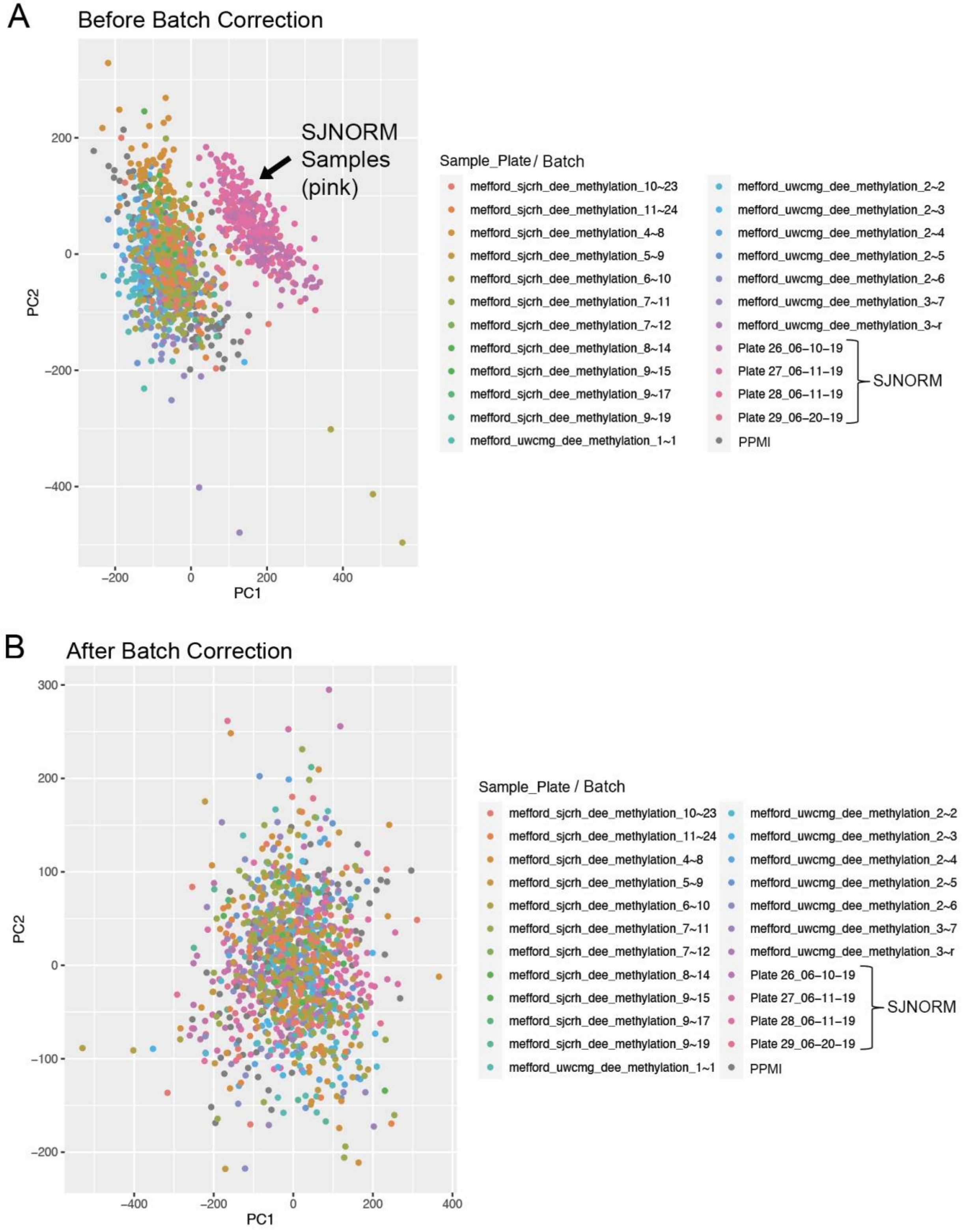
A
Before Batch Correction
SJNORM Samples (pink)
PC1
PC2
Sample_Plate / Batch
mefford_sjcrh_dee_methylation_10~23
mefford_sjcrh_dee_methylation_11~24
mefford_sjcrh_dee_methylation_4~8
mefford_sjcrh_dee_methylation_5~9
mefford_sjcrh_dee_methylation_6~10
mefford_sjcrh_dee_methylation_7~11
mefford_sjcrh_dee_methylation_7~12
mefford_sjcrh_dee_methylation_8~14
mefford_sjcrh_dee_methylation_9~15
mefford_sjcrh_dee_methylation_9~17
mefford_sjcrh_dee_methylation_9~19
mefford_uwcmg_dee_methylation_1~1
mefford_uwcmg_dee_methylation_2~2
mefford_uwcmg_dee_methylation_2~3
mefford_uwcmg_dee_methylation_2~4
mefford_uwcmg_dee_methylation_2~5
mefford_uwcmg_dee_methylation_2~6
mefford_uwcmg_dee_methylation_3~7
mefford_uwcmg_dee_methylation_3~r
Plate 26_06−10−19
Plate 27_06−11−19
Plate 28_06−11−19
Plate 29_06−20−19
SJNORM
PPMI
B
After Batch Correction
PC1
PC2
Sample_Plate / Batch
mefford_sjcrh_dee_methylation_10~23
mefford_sjcrh_dee_methylation_11~24
mefford_sjcrh_dee_methylation_4~8
mefford_sjcrh_dee_methylation_5~9
mefford_sjcrh_dee_methylation_6~10
mefford_sjcrh_dee_methylation_7~11
mefford_sjcrh_dee_methylation_7~12
mefford_sjcrh_dee_methylation_8~14
mefford_sjcrh_dee_methylation_9~15
mefford_sjcrh_dee_methylation_9~17
mefford_sjcrh_dee_methylation_9~19
mefford_uwcmg_dee_methylation_1~1
mefford_uwcmg_dee_methylation_2~2
mefford_uwcmg_dee_methylation_2~3
mefford_uwcmg_dee_methylation_2~4
mefford_uwcmg_dee_methylation_2~5
mefford_uwcmg_dee_methylation_2~6
mefford_uwcmg_dee_methylation_3~7
mefford_uwcmg_dee_methylation_3~r
Plate 26_06−10−19
Plate 27_06−11−19
Plate 28_06−11−19
Plate 29_06−20−19
SJNORM
PPMI

Supplementary Figure 1. Correction of DNA methylation array batch effects detected through PCA analysis. **A**. principal component analysis (PCA) plot using β values for probes located on chromosome (chr) 1 for all samples (n=1238 methylation array samples) after filtering out n=21 PCA outlier samples. Sample plate/batch (also listed in Supplementary Data 1B and 1C) labeled as “mefford” denote methylation arrays were performed at the UW or SJCRH for individuals with DEEs and controls as described. SJNORM plates indicate methylation arrays run for presumably healthy controls at SJCRH as a part of the SJLIFE study. A clear batch effect on PC1 is evident between the SJNORM samples and the rest of the samples included in this study. Although controls used from the PPMI study were run at a different institution, there is no batch effect with the PPMI cohort and our other samples. **B**. PCA plot after performing ComBat analysis, as detailed in the methods, where it is evident that the batch effect is eliminated. As a validation, DMR outlier analysis was performed (1) with batch correction of SJNORM as displayed and (2) with batch correction accounting for every batch as shown in the figure legend. Method (1) was chosen as the best approach, since method (2) resulted in the overcorrection and the loss of a significant disease-causing DMR with a robustly underlying DNA defect.

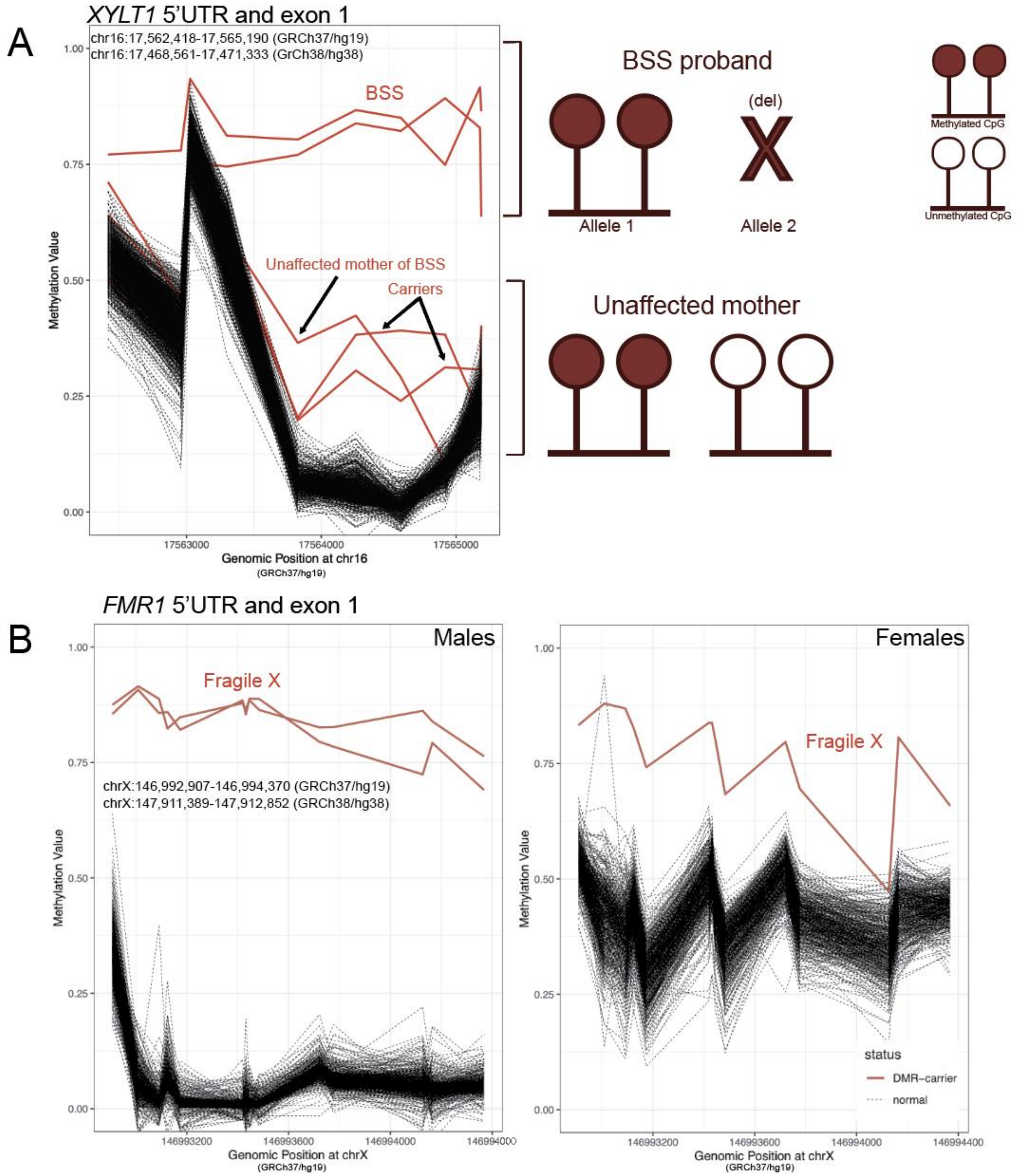

Supplementary Figure 2. DMR plots from rare outlier analysis of known hypermethylation events. **A.** DMR plot of the *XYLT1* 5'UTR and exon 1 showing two individuals with Baratela-Scott syndrome (BSS) with hemizygous hypermethylation (red) around β=~1 compared to controls (black). Each inflection point corresponds to an individual CpG probe from the array. These individuals with BSS harbor hypermethylation of *XYLT1* on one allele and a deletion

encompassing this region on the other, represented in the schematic to the right. One unaffected mother and two other carriers (red) carry heterozygous hypermethylation around β=0.5. **B.** DMR plots of the *FMR1* 5'UTR and exon 1 with individuals split by males (left) and females (right) for targeted sex chromosome outlier DMR analysis. Fragile X males and females are clearly hypermethylated (β=~1) compared to their counterpart controls.

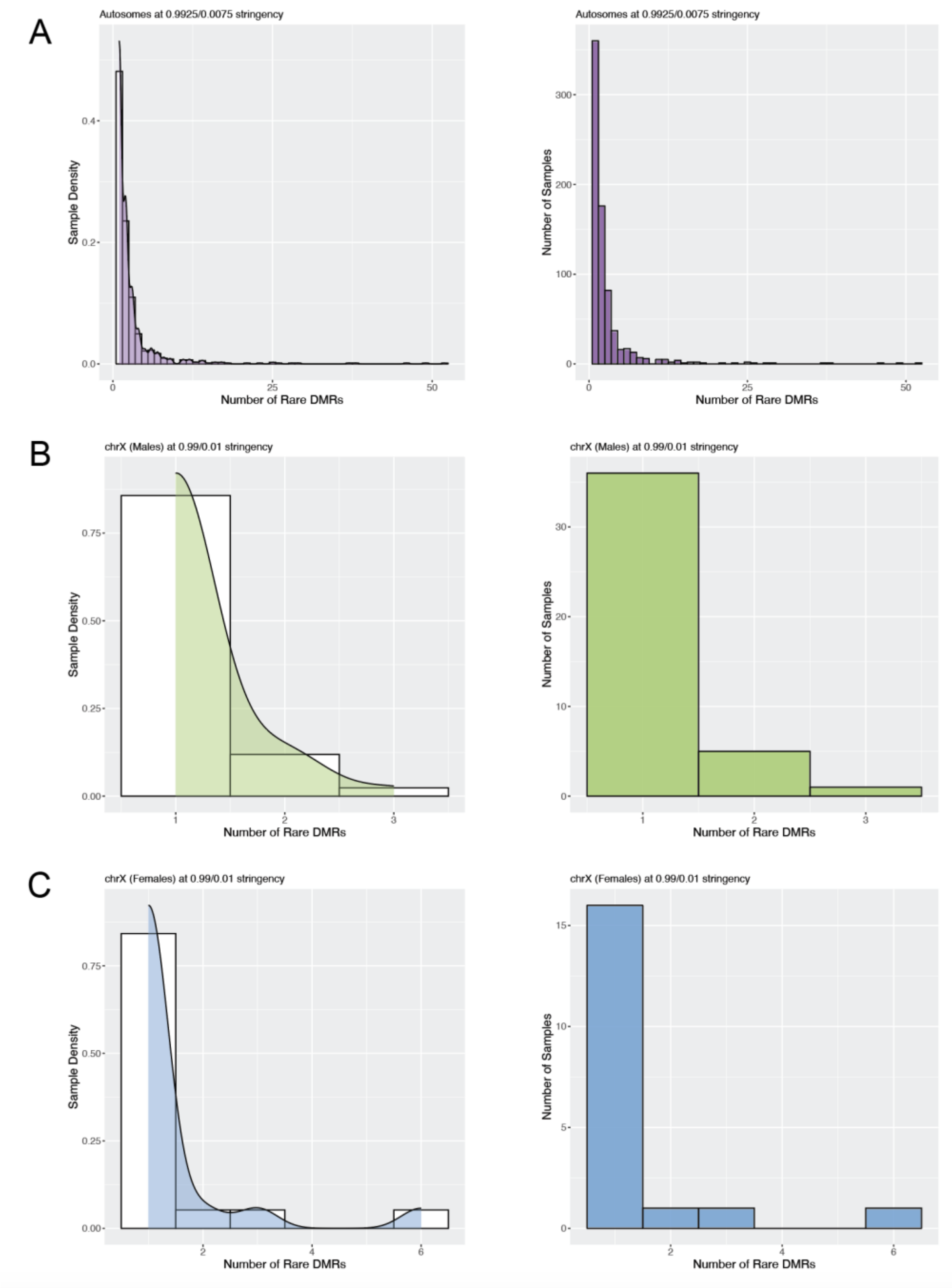

Supplementary Figure 3. Distribution of outlier DMRs across samples. Distribution plots showing sample density (left) and sample counts (right). **A.** DMRs called on autosomes (n=2,185) are in purple for the cohort (n=1,221 array samples analyzed after positive control samples n=3 LCL and n=2 saliva removed from final analysis), **B.** DMRs called on chrX (n=49) for male samples (n= 652 array samples analyzed) are in green, and **C.** DMRs called on chrX

(n=27) for female samples (n= 569 array samples analyzed) are in blue. In all cases, the majority of samples carry only 1 DMR, indicating that the outlier criteria were successful in identifying rare DMRs.

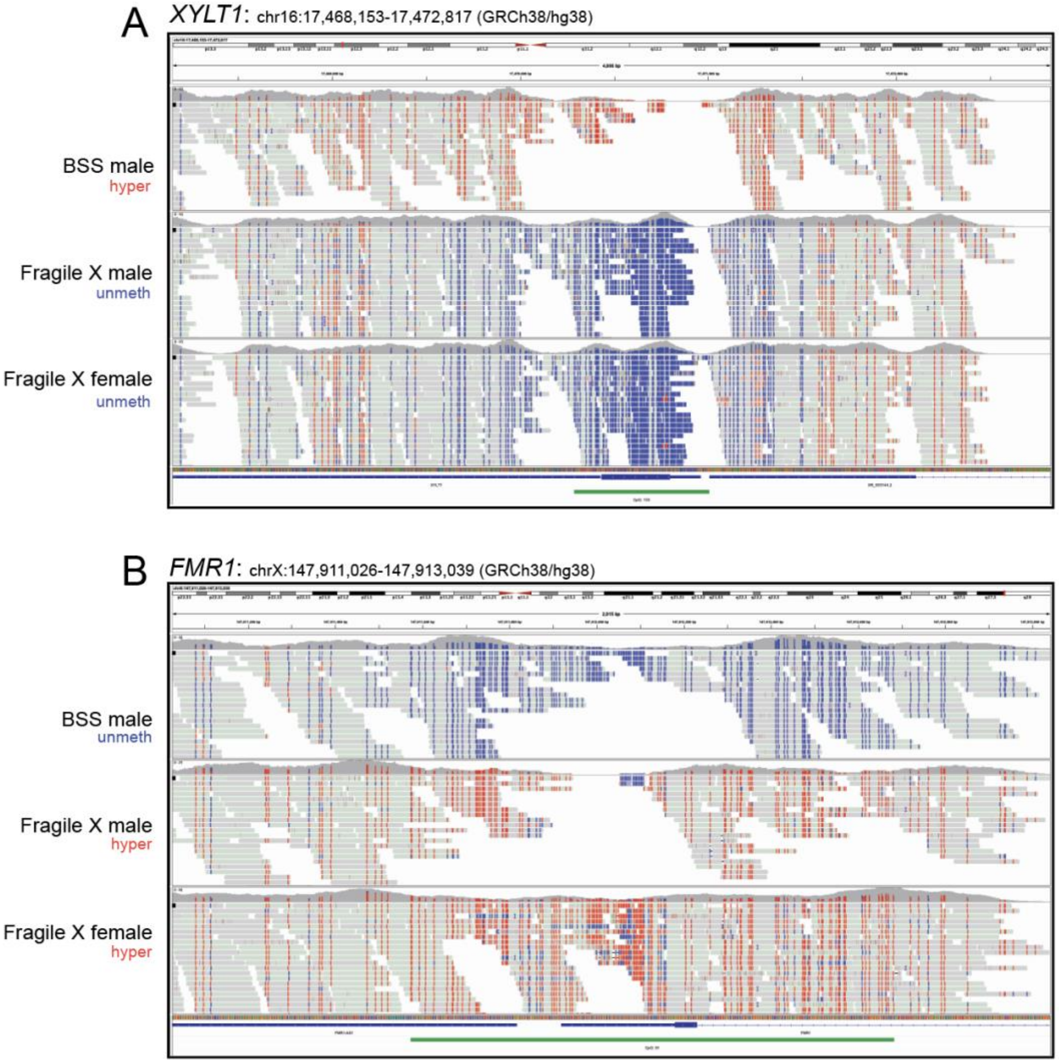

Supplementary Figure 4. Validation of known outlier DMRs using targeted EM-seq. **A.** IGV view of targeted EM-seq reads at the *XYLT1* 5'UTR and exon 1 that are hypermethylated in an individual with BSS (upper) compared to methylation-negative controls (middle and lower). **B.** IGV screenshot of targeted EM-seq reads at the *FMR1* 5'UTR and exon 1 hypermethylated in Fragile X male and female individuals (middle and lower) compared to methylation-negative control (upper).

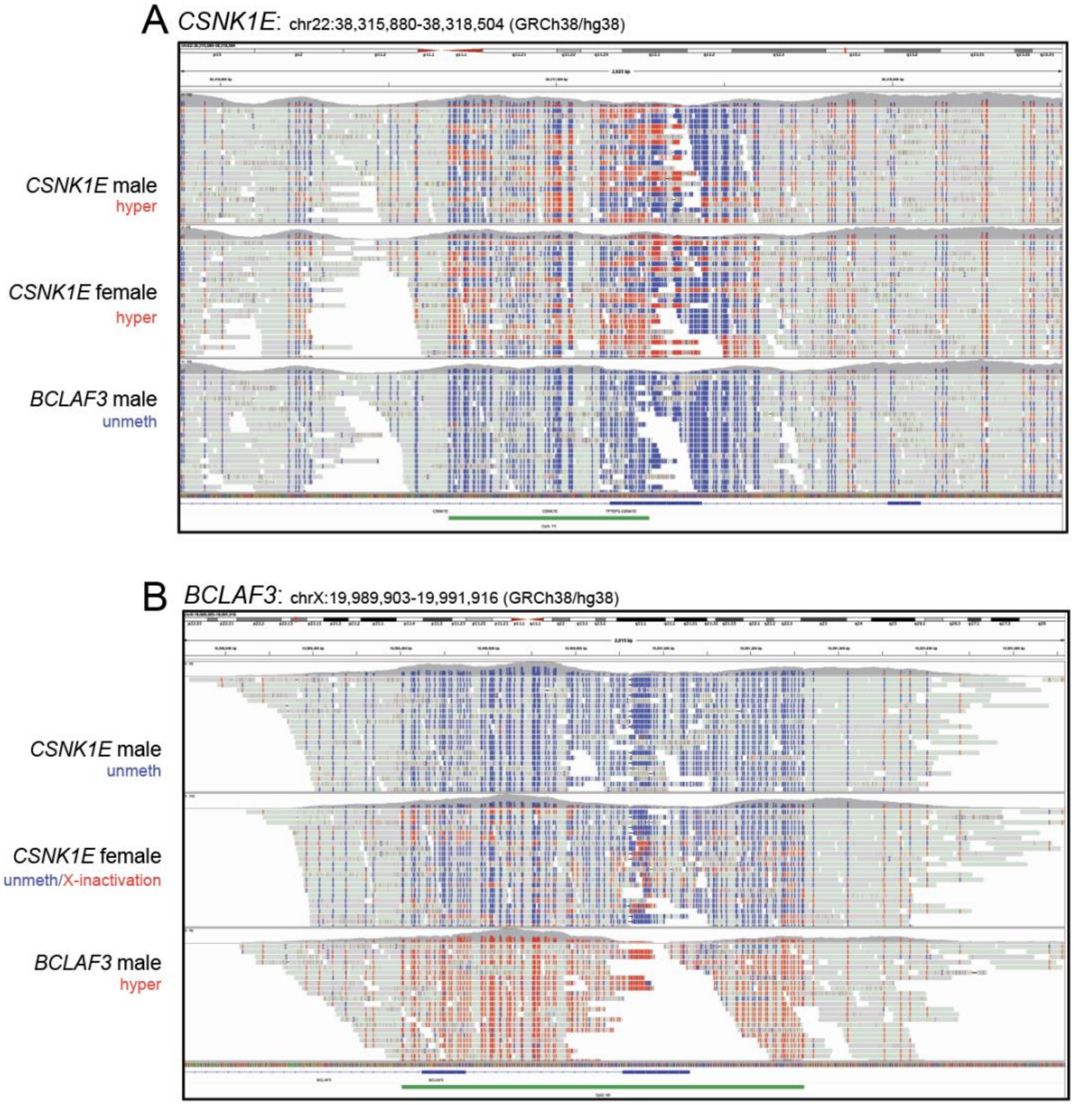

Supplementary Figure 5. Validation of rare outlier DMRs called from methylation array in individuals with unsolved DEEs using targeted EM-seq. **A.** IGV view of *CSNK1E* 5'UTR and intron 1 validating heterozygous hypermethylation in two individuals with unsolved DEEs (upper and middle) compared to methylation-negative control (lower). **B.** IGV view of *BCLAF3* 5'UTR validating hemizygous hypermethylation called from methylation array in a male individual with unsolved DEEs (lower) compared to methylation-negative controls (upper and middle).

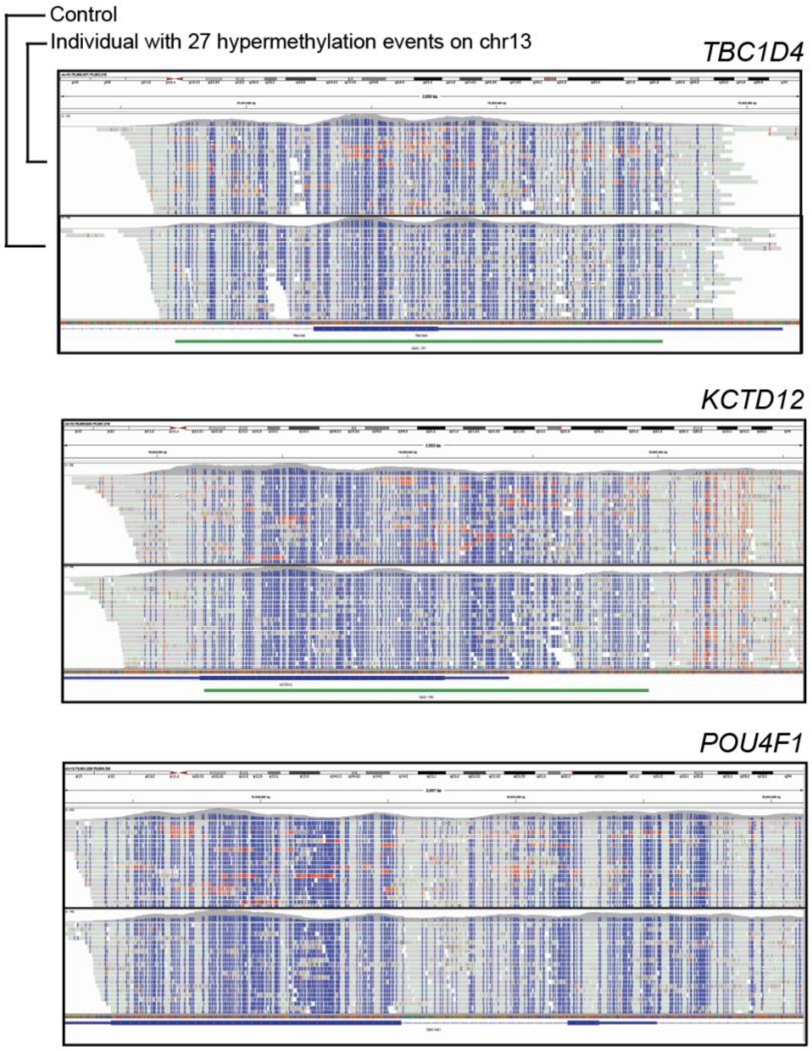

Supplementary Figure 6. Validation of rare outlier DMRs on chr13 in a single individual using targeted EM-seq. Representative images depict IGV views for 3/26 DMRs on chr13 for an individual with unsolved DEE (upper panel of each box) compared to a control (lower panel of each box).

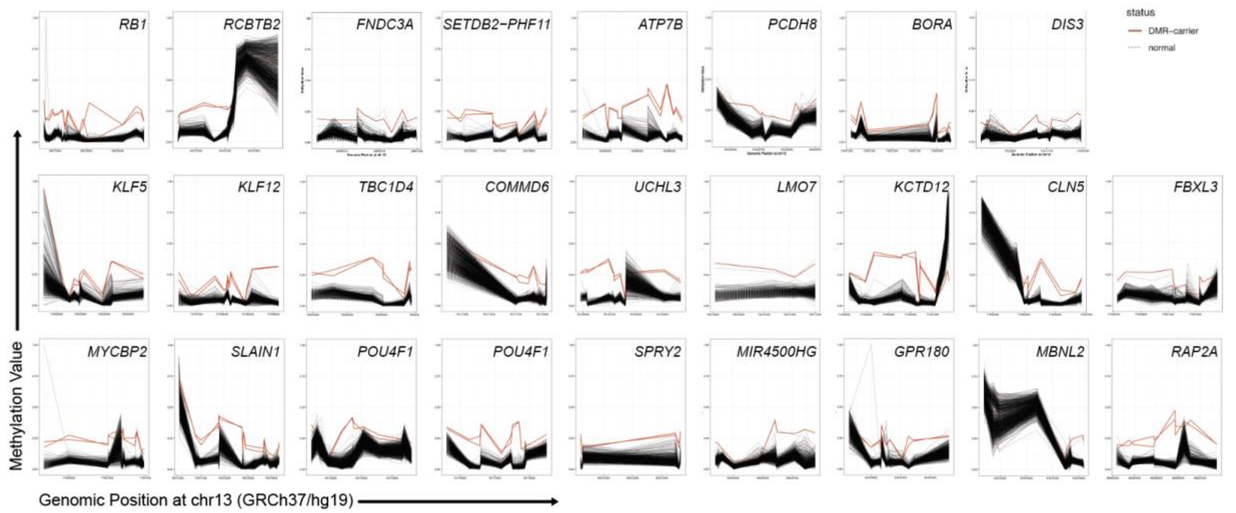

Supplementary Figure 7. Individual with unsolved DEE displaying multiple hypermethylated DMRs across chr13. DMR plots depicting DNA methylation array data are shown for all n=26 outlier DMRs called for an individual with unsolved DEE (red) compared to controls (black).

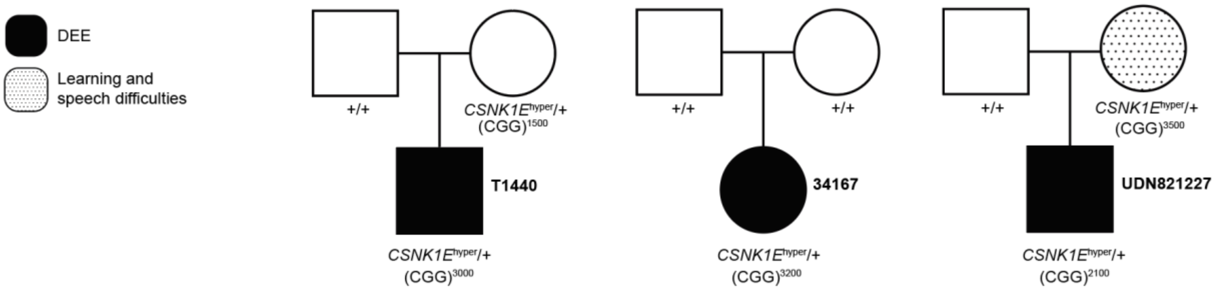

	Family 1	Family 2	Family 3
Methylation array (Blood)	Maternally inherited hypermethylation	*de novo* hypermethylation	Maternally inherited hypermethylation
Long-read sequencing (Blood)	1,500-3,000bp CGG repeat expansion in the proband inherited from mother (~1,500bp)	1,100-3,200bp CGG repeat expansion in the proband	1,300-2,100bp CGG repeat expansion in proband inherited from mother (~270-3,500)
RNA-seq (Fibroblasts)	NA	Decreased gene expression in proband	Decreased gene expression in proband and mother

Supplementary Figure 8. Family pedigrees for a cohort of individuals with unsolved DEEs displaying hypermethylation of the 5'UTR and exon 1 of *CSNK1E*. Pedigrees show the inheritance patterns of *CSNK1E* hypermethylation and affected vs. unaffected status across the families. The lower table shows the data types analyzed from each family and a summary of any associated results. The schematics shown on the left panel were created with BioRender.com and released under a Creative Commons Attribution-Non-Commercial-Noderivs 4.0 International License (CC-BY-NC-ND).

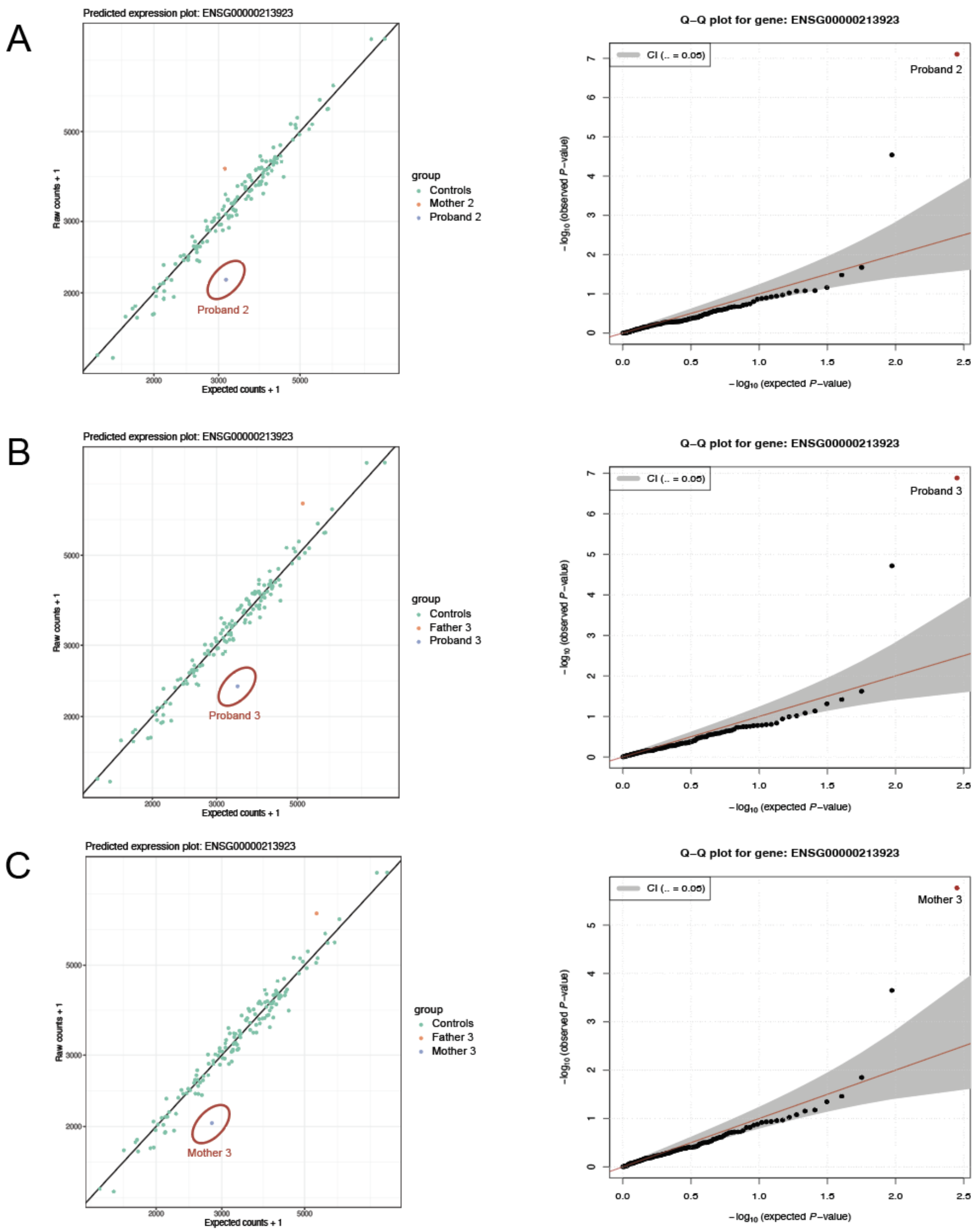

Supplementary Figure 9. Dropout Analysis of RNA-seq data for *CSNK1E* from human-derived fibroblasts by family. OUTRIDER predicted expression plot is shown on the left and Q-Q plot on the right. **A**. Proband 2 "drops out" for *CSNK1E* expression (*padj*=0.00112276, zScore=-5.63, $\log_2$FC=-0.55) compared to Mother 2 and control fibroblast cohort of 139 publicly available

samples[31]. **B**. Proband 3 “drops out” for *CSNK1E* expression compared to Father 3 and the control fibroblast cohort (*padj*=0.00228623, zScore=-5.54, log_2FC=-0.53). **C**. Mother 3 “drops out” for *CSNK1E* expression compared to Father 3 and the control fibroblast cohort (*padj*=0.021391407, zScore=-5.02, log_2FC=-0.47).

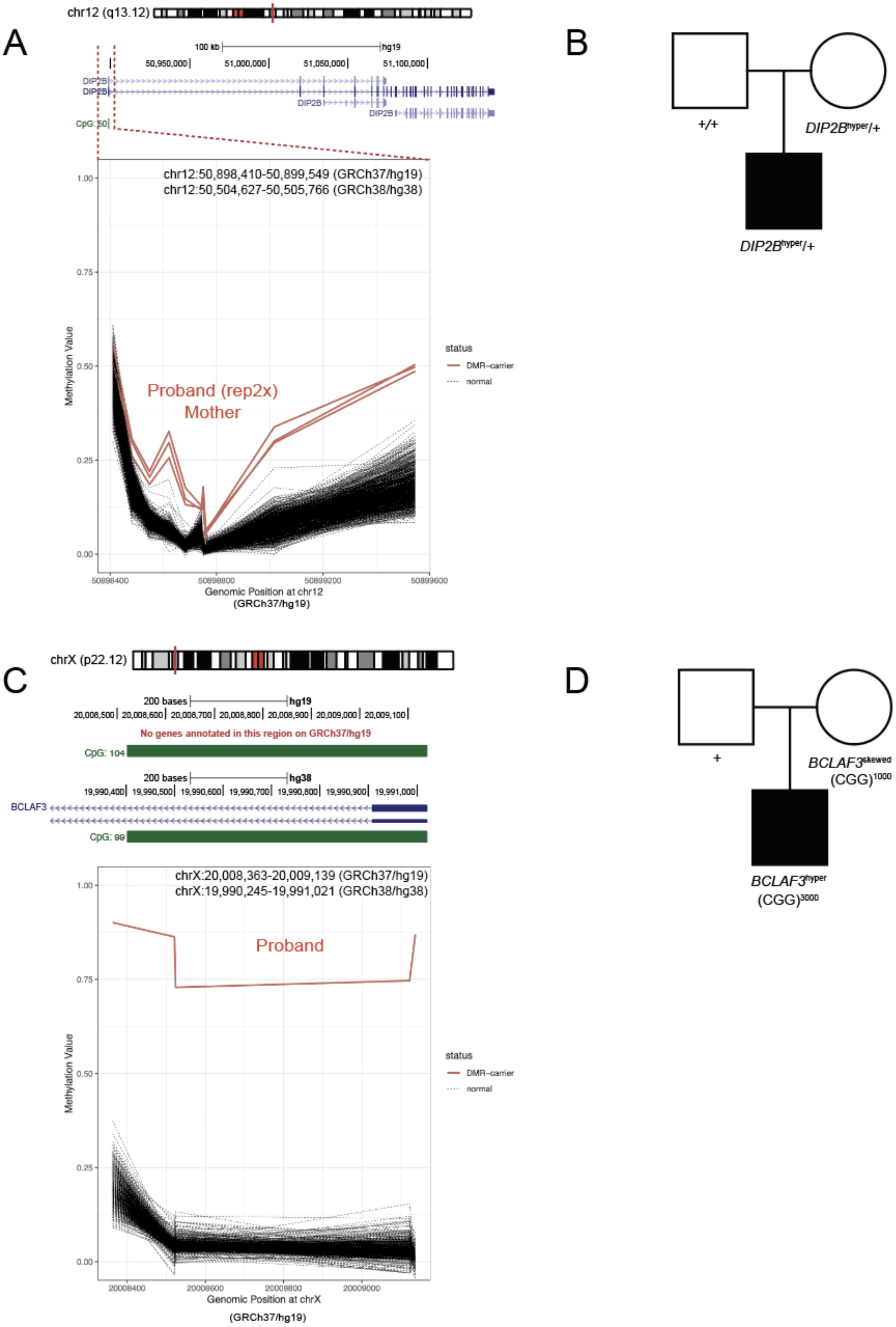

A
chr12 (q13.12)
100 kb
hg19
50,950,000
51,000,000
51,050,000
51,100,000
DIP2B
DIP2B
DIP2B
DIP2B
CpG: 50
chr12:50,898,410-50,899,549 (GRCh37/hg19)
chr12:50,504,627-50,505,766 (GRCh38/hg38)
Methylation Value
Proband (rep2x)
Mother
status
DMR-carrier
normal
Genomic Position at chr12
(GRCh37/hg19)
B
+/+
DIP2B hyper/+
DIP2B hyper/+
C
chrX (p22.12)
200 bases
hg19
No genes annotated in this region on GRCh37/hg19
CpG: 104
200 bases
hg38
BCLAF3
CpG: 99
chrX:20,008,363-20,009,139 (GRCh37/hg19)
chrX:19,990,245-19,991,021 (GRCh38/hg38)
Methylation Value
Proband
status
DMR-carrier
normal
Genomic Position at chrX
(GRCh37/hg19)
D
+
BCLAF3 skewed
(CGG)1000
BCLAF3 hyper
(CGG)3000

Supplementary Figure 10. Outlier hypermethylation of regions underlying known and novel repeat expansions. **A**. DMR plot for outlier hypermethylated DMR at the *DIP2B* promoter and exon 1 in a proband with unsolved DEE (repeated twice on the methylation array) and his mother (both in red). **B**. Pedigree showing inheritance pattern of the *DIP2B* DMR. **C**. DMR plot of targeted X chromosome analysis in males depicting outlier hypermethylated DMR at an intergenic region (by GRCh37/hg19 annotation) and exon 1 of an uncharacterized gene *BCLAF3* (by GRCh38/hg38 annotation) in a male proband with unsolved DEE. **D**. Pedigree shows CGG *BCLAF3* repeat expansion is inherited from the mother and expanded in the proband. Data for the repeat expansion are shown in Figure S11.

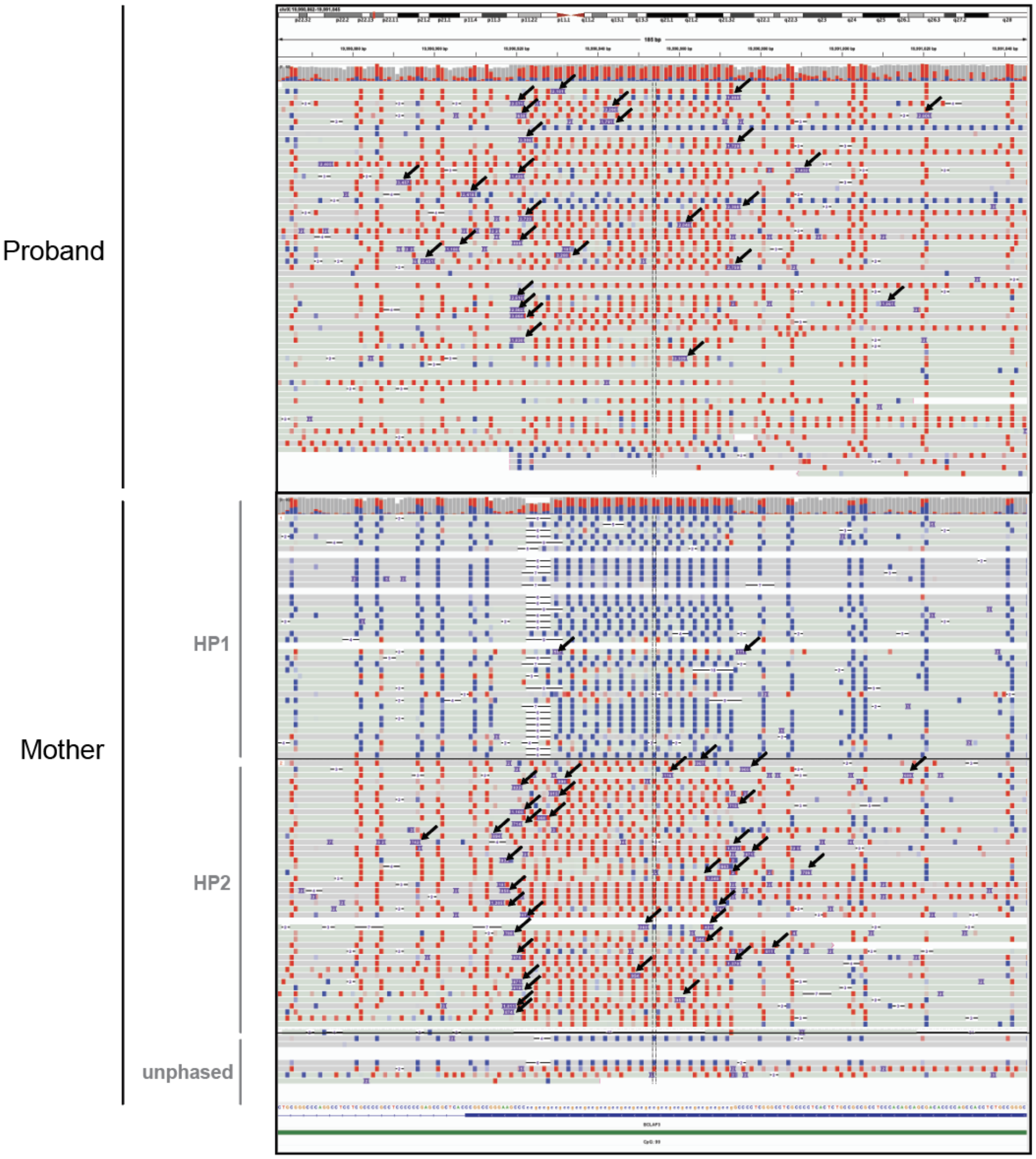

Supplementary Figure 11. Long-read sequencing identifies a novel repeat expansion at the 5'UTR of *BCLAF3*. ONT long-read sequencing reads in IGV phased for 5mC for the proband validating *BCLAF3* hypermethylation (top panel). Long-read sequencing further indicates a novel ~2,500-3,000bp CG-rich repeat expansion (arrows). Haplotype resolved ONT long-read sequencing reads in IGV for the proband's mother (bottom panel) displaying smaller ~700-1,000bp CG-rich repeat expansion (arrows). Haplotype resolution also indicates that the mother's

X-chromosome inactivation is skewed against the repeat allele, not a random 50/50 hypermethylation across both X chromosomes.

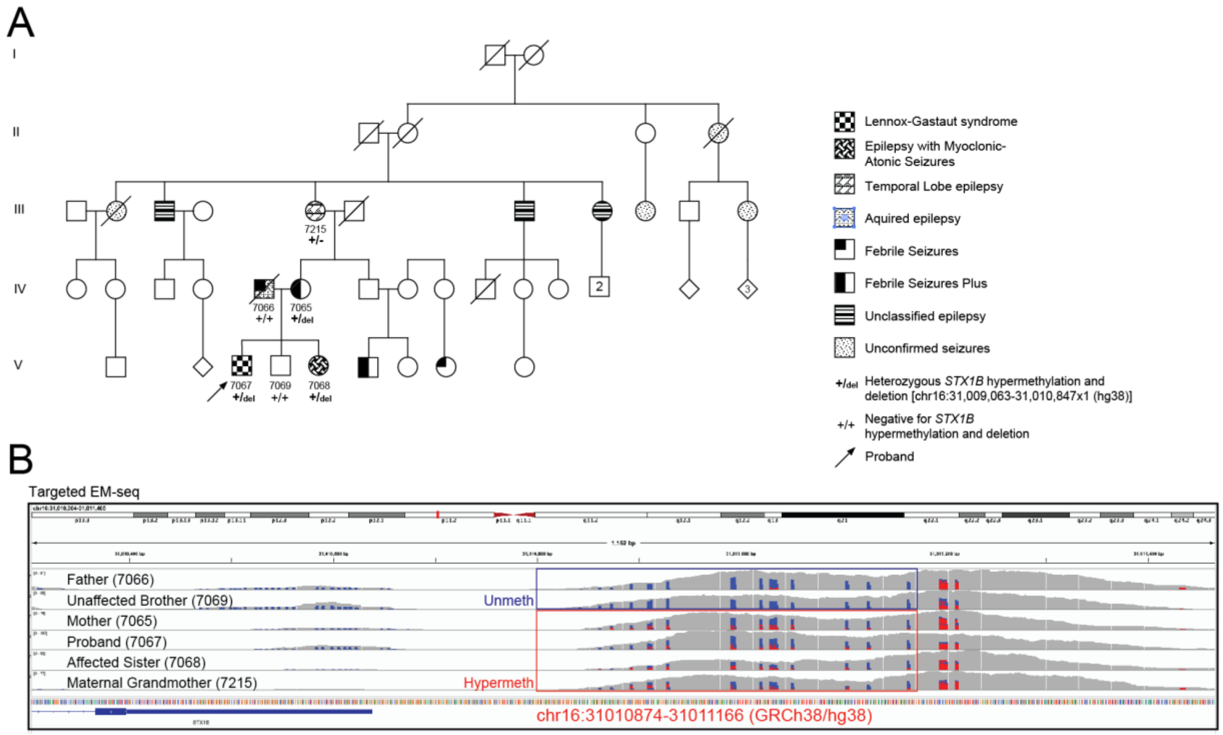

Supplementary Figure 12: Hypermethylation of the *STX1B* TSS and promoter validated by targeted EM-seq in a family with GEFs+ phenotypes. **A**. Extended pedigree for the family of proband 7067 displaying related phenotypes throughout the maternal side and extended family. **B**. Coverage traces from targeted EM-seq data depict the proportion of methylated (red) and unmethylated (blue) CpGs in the *STX1B* DMR. Hypermethylation is validated, and boundaries of DNA methylation are extended in proband, affected sister, affected mother, and affected maternal grandmother. The unaffected brother and father are unmethylated.

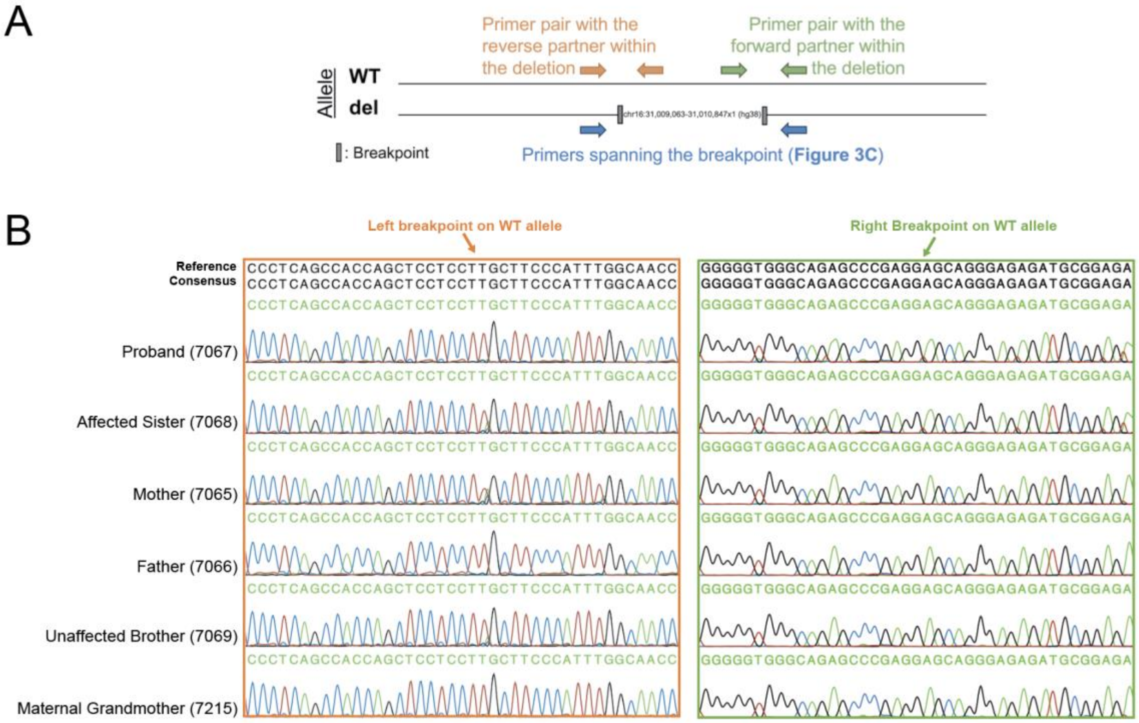

Supplementary Figure 13: A deletion encompassing intron 1, exon 1, the TSS, and the promoter of *STX1B* in a family with GEFs+ phenotypes. **A**. Schema of PCR validation strategy, including the wild-type (WT) and deletion (del) alleles. **B**. Control PCR showing the breakpoint regions on the WT allele (orange and green) where a PCR product is obtained for the unaffected brother and father. This contrasts with the PCR shown in Figure 3C, where no PCR product is obtained for the unaffected brother and father for the primer pair that spans the breakpoints, indicating that they each do not carry the deletion.

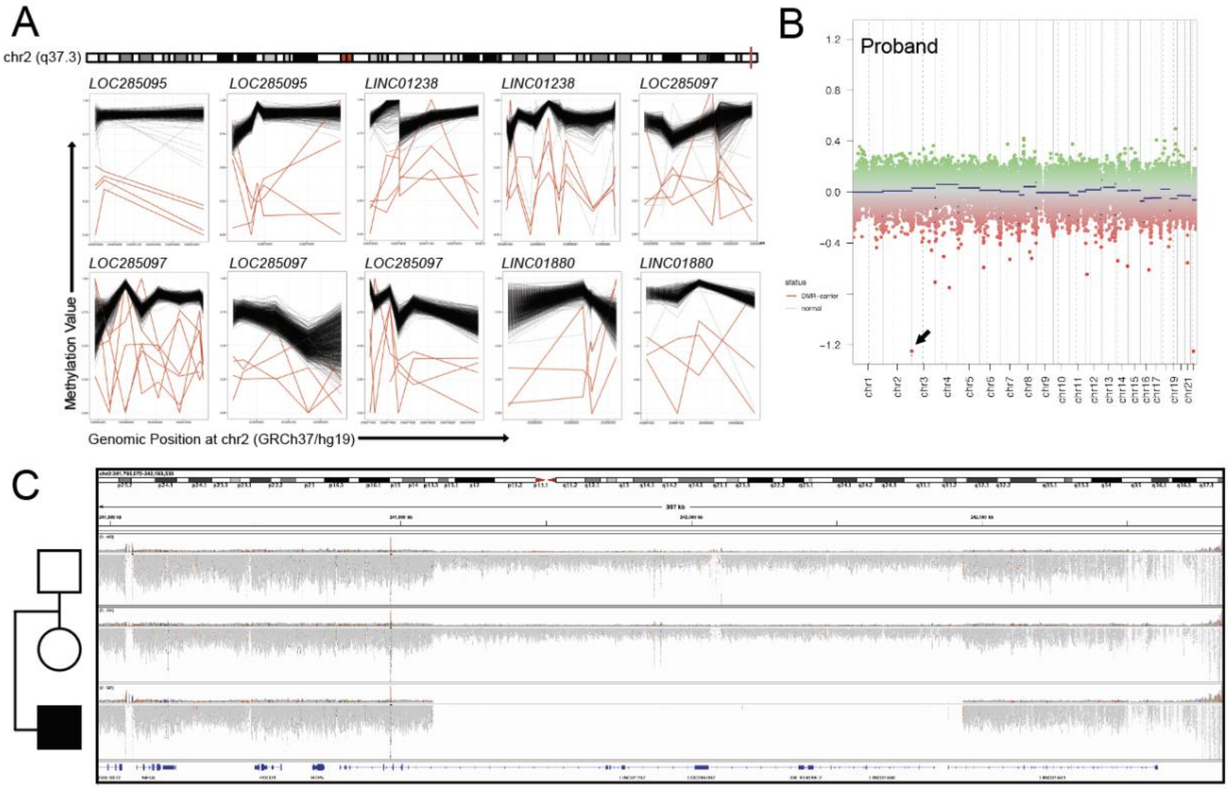

Supplementary Figure 14. Outlier "DMRs" at chr2 represent copy number deletion. **A**. DMR plots showing 10 representative "hypomethylated DMRs" across 2q37.3 for an individual with unsolved DEE (run in triplicate, red) and one control (red). **B**. CNV calling from the array with the R package "conumee" detects copy number deletion overlapping with chr2 DMR locus in individual with unsolved DEE. **C**. GS of trio identifies inherited homozygous copy number deletion in proband.

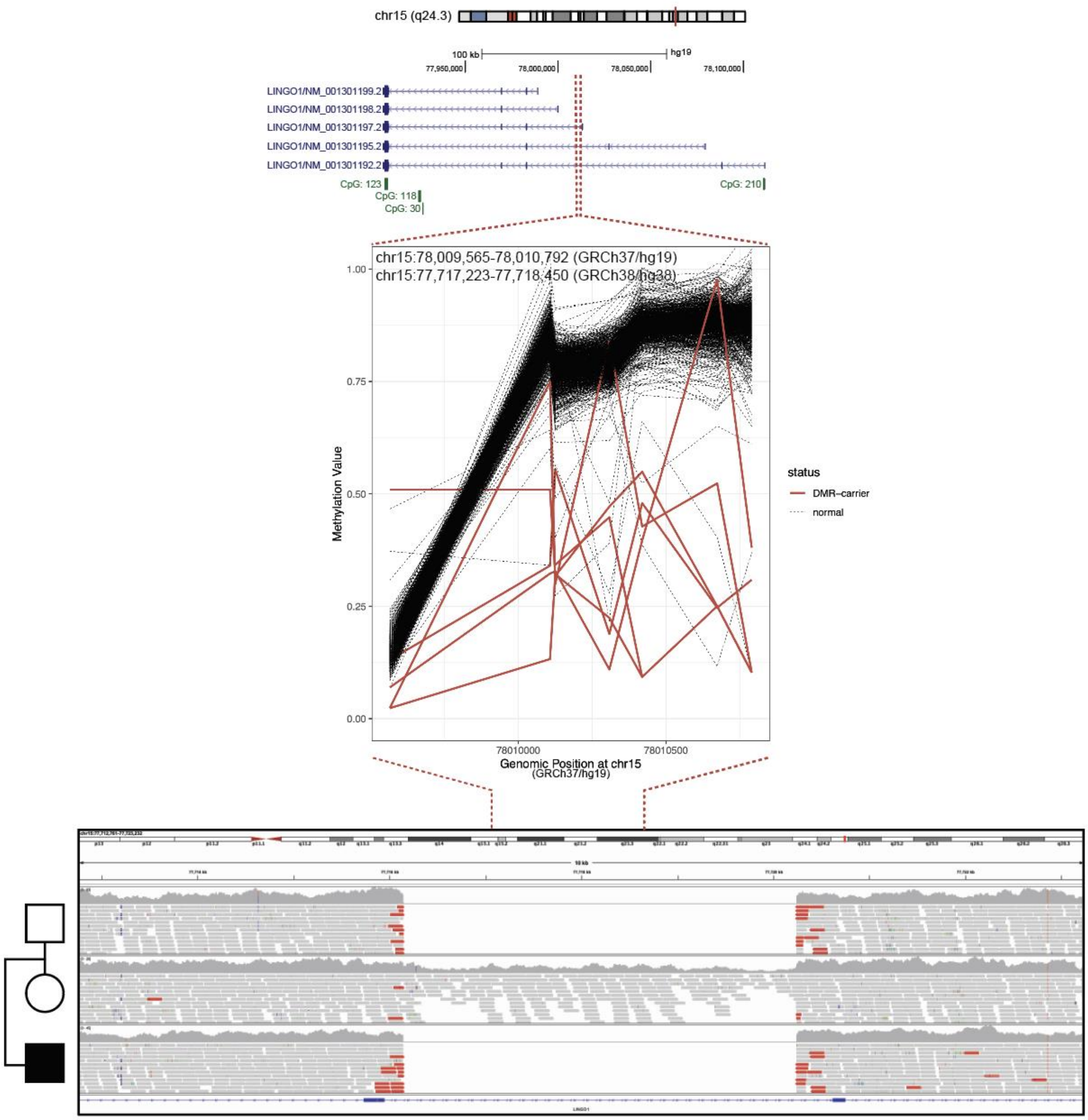

Supplementary Figure 15. Outlier "DMR" at *LINGO1* intron represents copy number deletion. Annotation track showing the location of DMR in different *LINGO1* transcripts (upper). DMR plot showing hypomethylated DMR detected in several DMR carriers, including a proband with two replicates and a mother. GS detects inherited copy number deletion in the proband (lower).

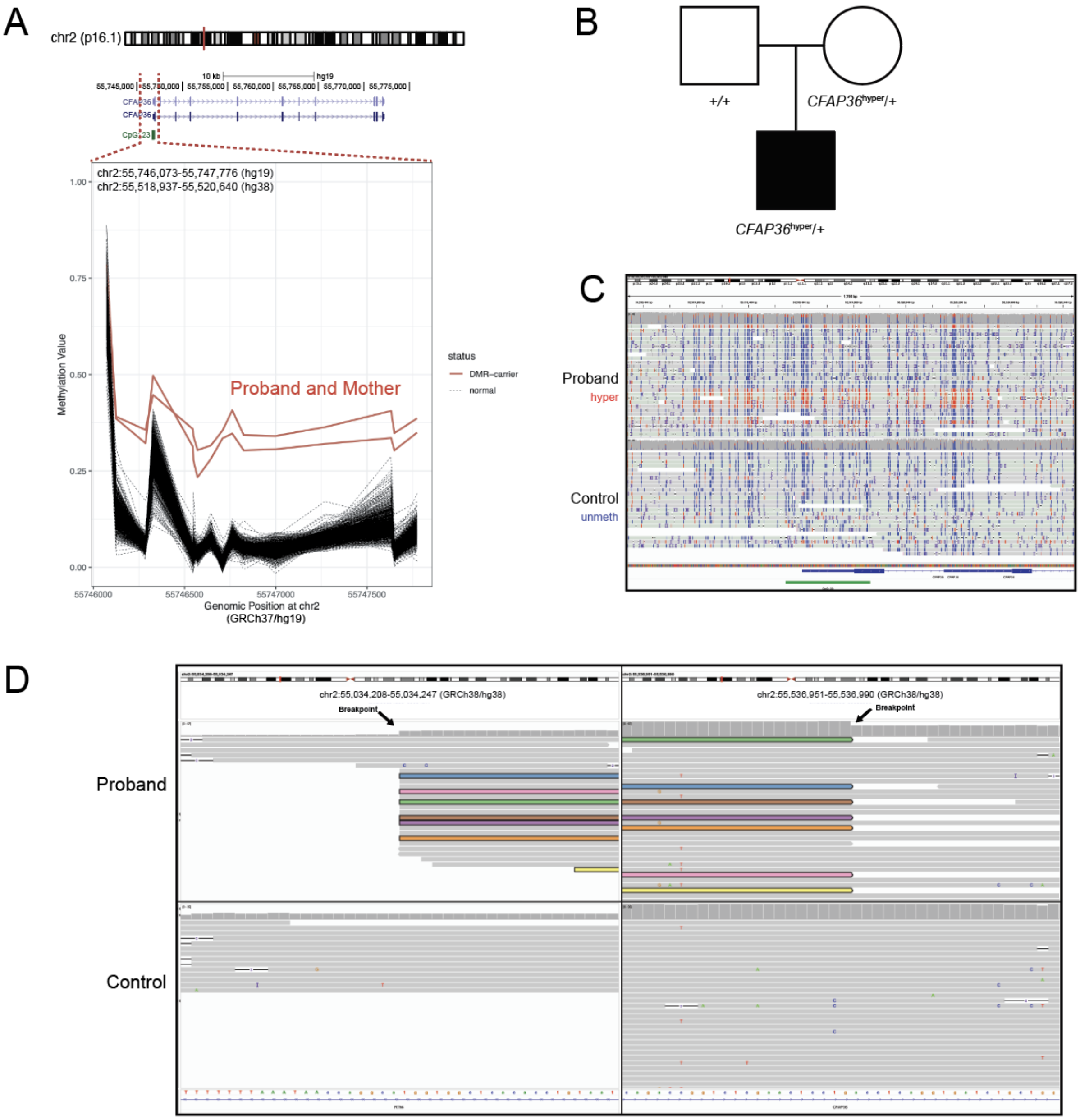

Supplementary Figure 16. Outlier hypermethylated DMR at *CFAP36* due to a tandem duplication. **A**. DMR plot showing hypermethylation at *CFAP36* in a proband with unsolved DEE and mother. **B**. Pedigree depicting inheritance pattern of DMR. **C**. IGV screenshot of targeted ONT long reads phased for 5mC at DMR region validating hypermethylation called from the array. **D**. IGV screenshot showing the location of the breakpoints of the tandem duplication and associated "dip" in coverage. Some but not all the reads showing the breakpoint are colored. The

upstream breakpoint (left) was not included in the adaptive sampling target region and therefore has lower coverage compared to the target region.

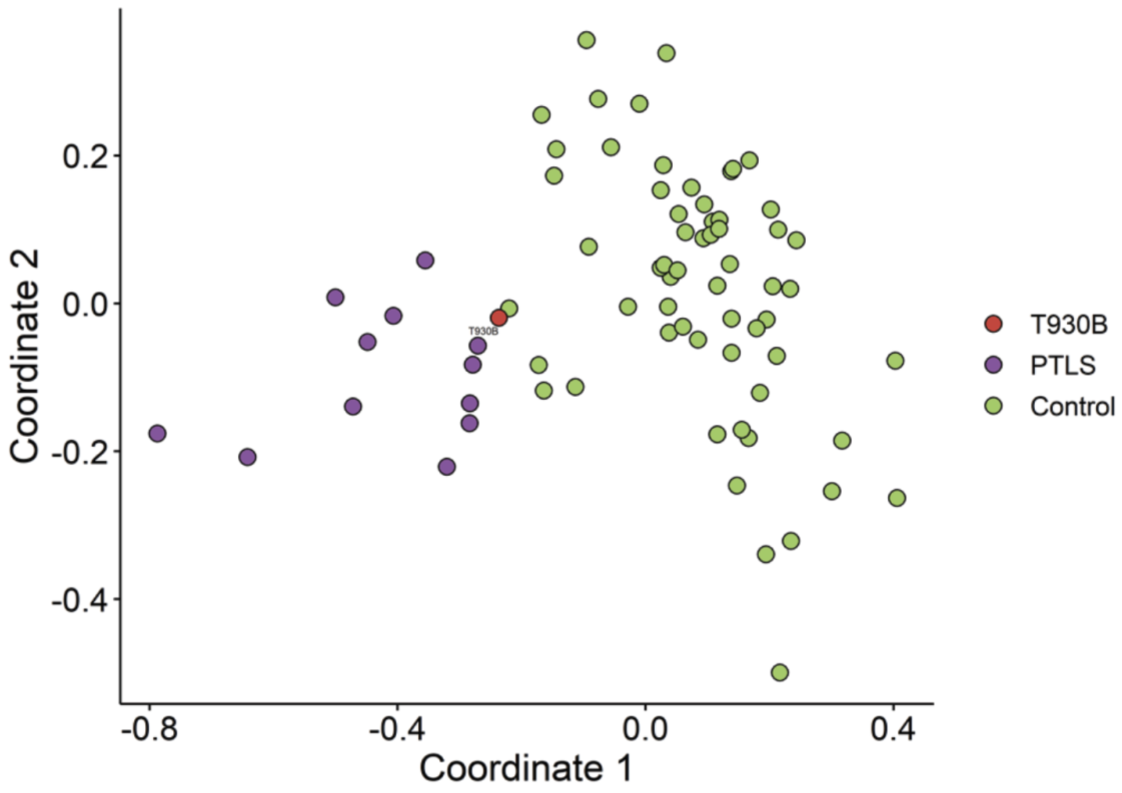

Supplementary Figure 17. Inconclusive episignature results. An example of inconclusive results for Potocki-Lupski syndrome (PTLS) episignature. Individual T930B (red) was reported as inconclusive for PTLS due to a low MVP score of 0.053 and inconsistent MDS clustering between cases (purple) and unaffected controls (green). Inconclusive episignature results are reported with the caveat that further follow up or investigation may be warranted if there is a clinical phenotype consistent with the inconclusive episignature in question.

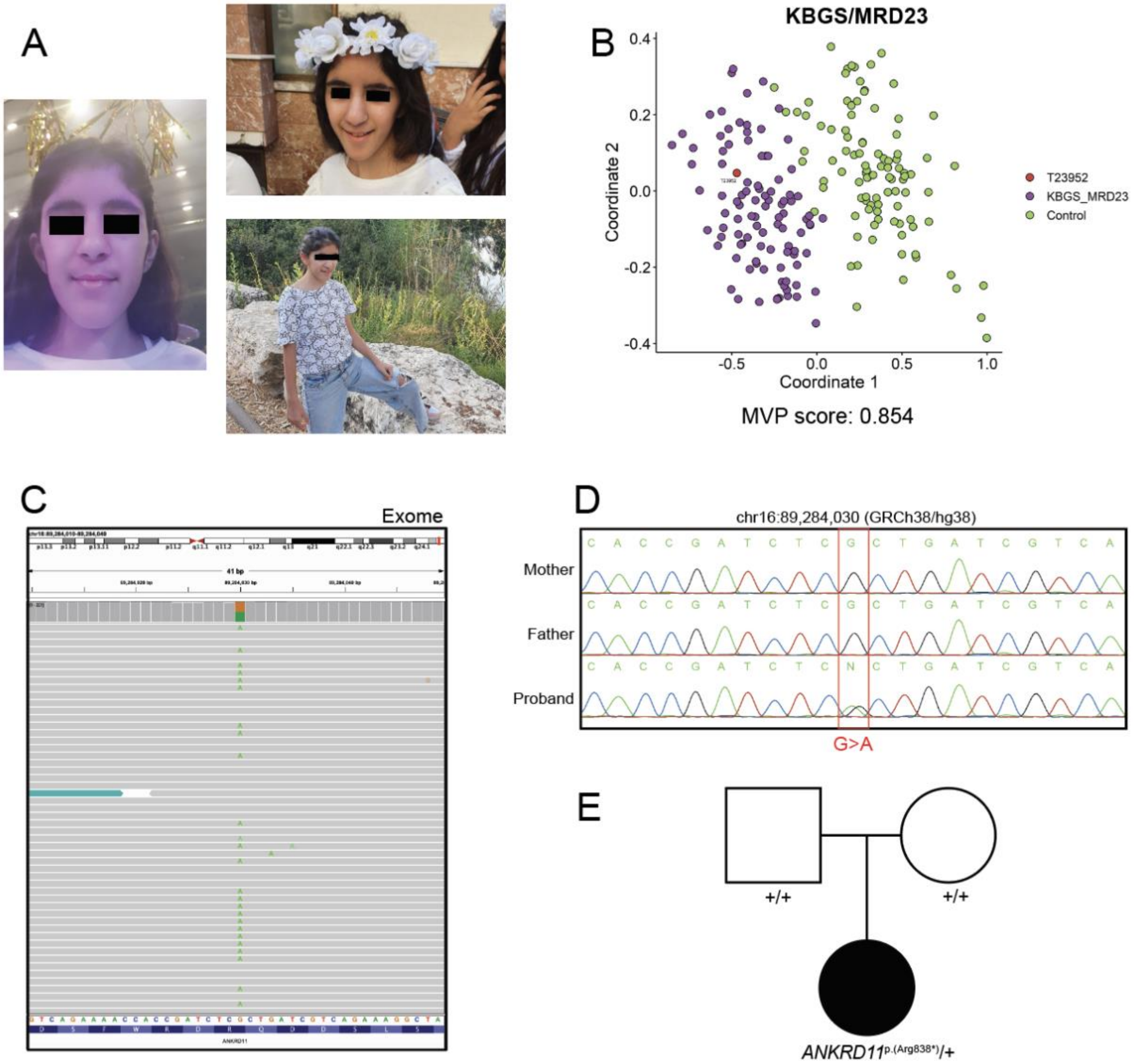

Supplementary Figure 18. Diagnosis of *ANKRD11* enabled by episignature screening. **A.** Photos of affected individual displaying dysmorphic features, including triangular face, bulbous nose, thin upper lip, featureless philtrum, broad bushy eyebrows, large prominent ears, thin upper lip. **B.** Multidimensional scaling (MDS) plot showing how proband (red) clusters with KBGS_MRD23 episignature (purple). **C.** IGV screenshot of a pathogenic variant in *ANKRD11* detected by exome sequencing. **D**. PCR validation and segregation of the variant. **E.** Pedigree indicating that the variant occurs *de novo*.

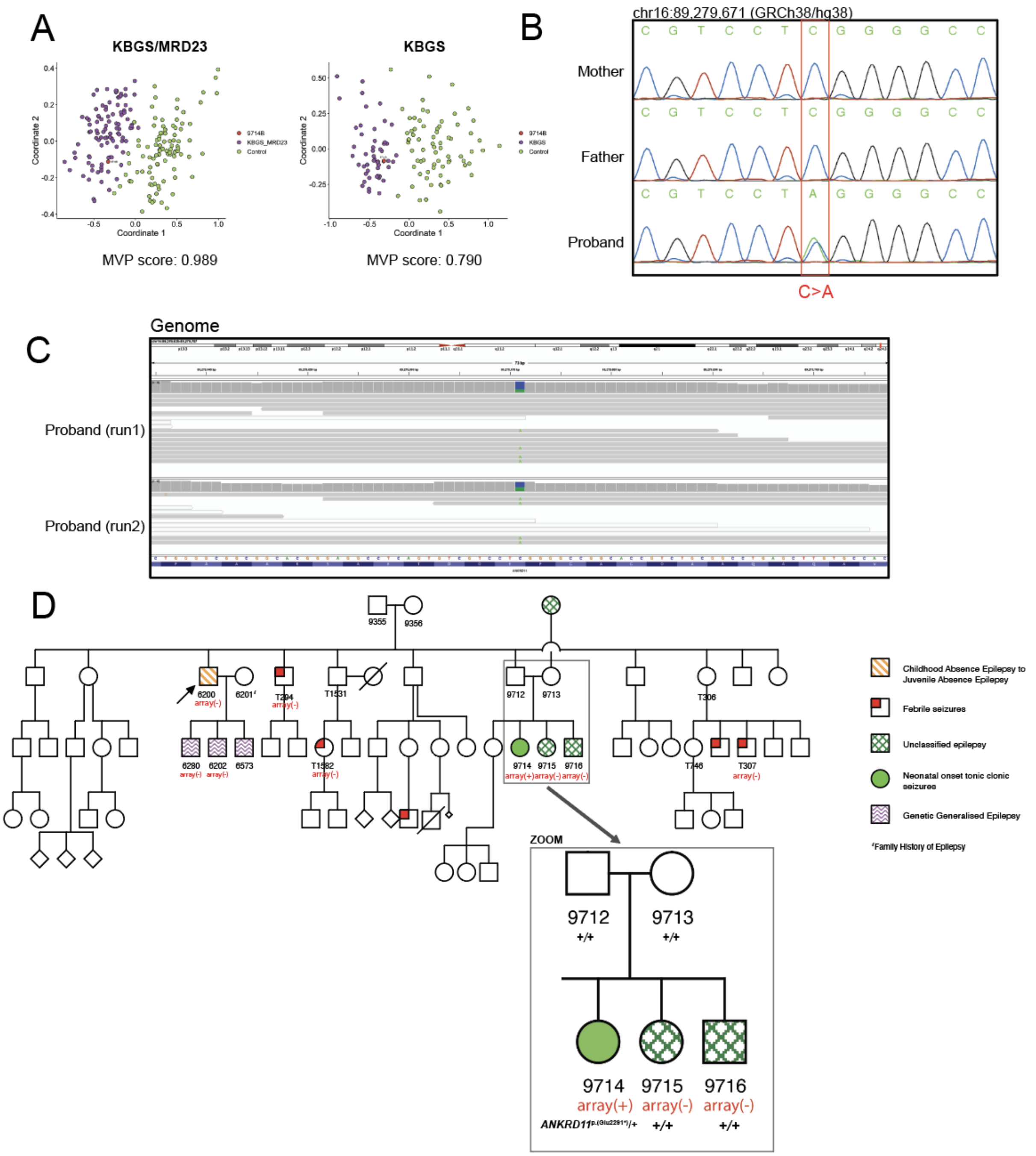

Supplementary Figure 19. Diagnosis of *ANKRD11* enabled by episignature screening. **A**. MDS plot showing proband (red) clustering with KBGS_MRD23 affected controls (purple, left) and further clustering with the secondary KBGS signature (purple, right). **B**. Sanger sequencing validation and segregation of pathogenic variant recovered from **C**. Two low coverage GS runs showing the presence of the pathogenic variant. **D**. Familial pedigree illustrating multiple

generations and manifestations of epilepsy. "Array" in red indicates that DNA methylation array on proband and additional affected family members (n=8). The red (+) indicates the individual was episignature positive, and (-) indicates episignature negative. Since the variant is *de novo* and not present in any of the tested affected family members, *ANRKRD11* variation is not the cause of the familial epilepsy but explains the more severe phenotype of 9714.

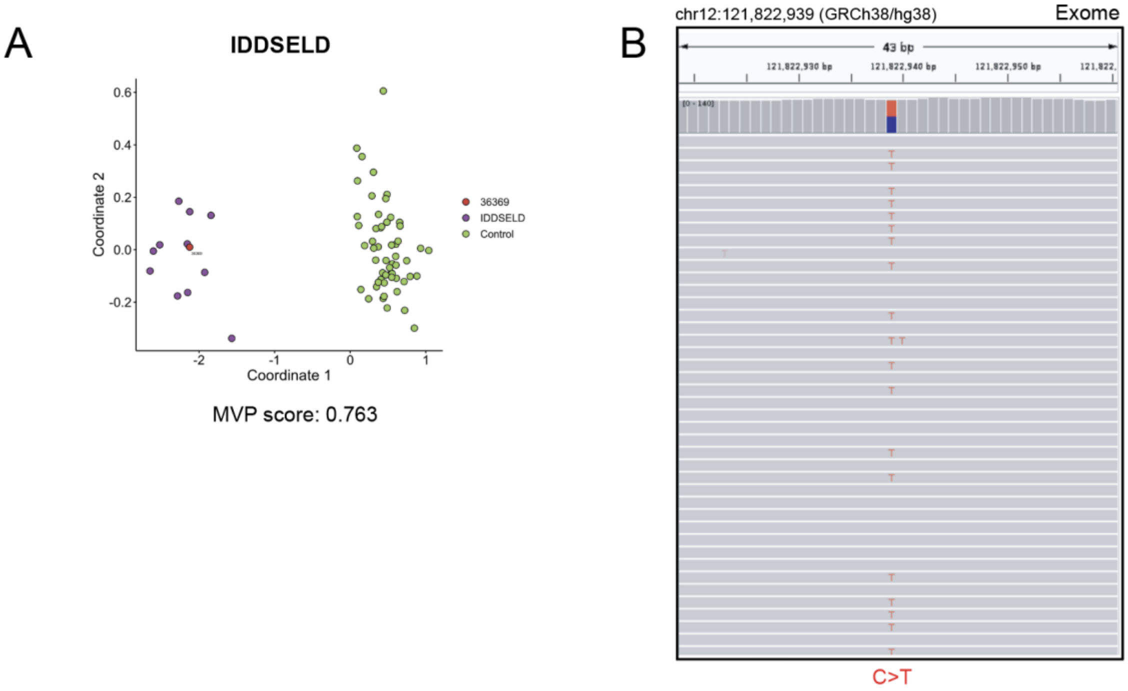

Supplementary Figure 20. Diagnosis of *SETD1B* enabled by episignature screening. **A**. MDS plot showing clustering of the proband (red) with the IDDSELD episignature (purple). **B**. IGV screenshot of pathogenic variant identified by exome sequencing.

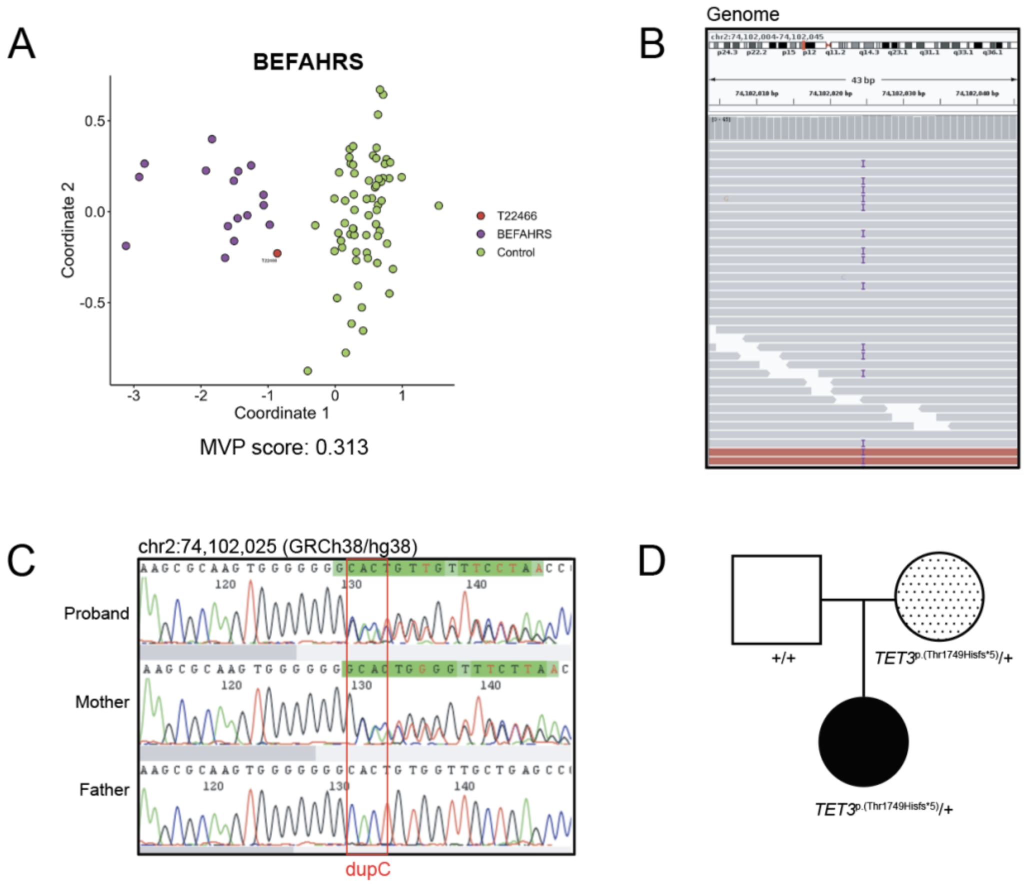

Supplementary Figure 21. Diagnosis of *TET3* enabled by episignature screening. **A**. MDS plot showing clustering of the proband with the BEFAHRS episignature. **B**. IGV screenshot of pathogenic duplication identified through GS. **C**. Sanger sequencing validation and segregation of variant indicating that **D**. the *TET3* variant is inherited from the mother, who is mildly phenotypically affected (Supplementary Phenotype data).

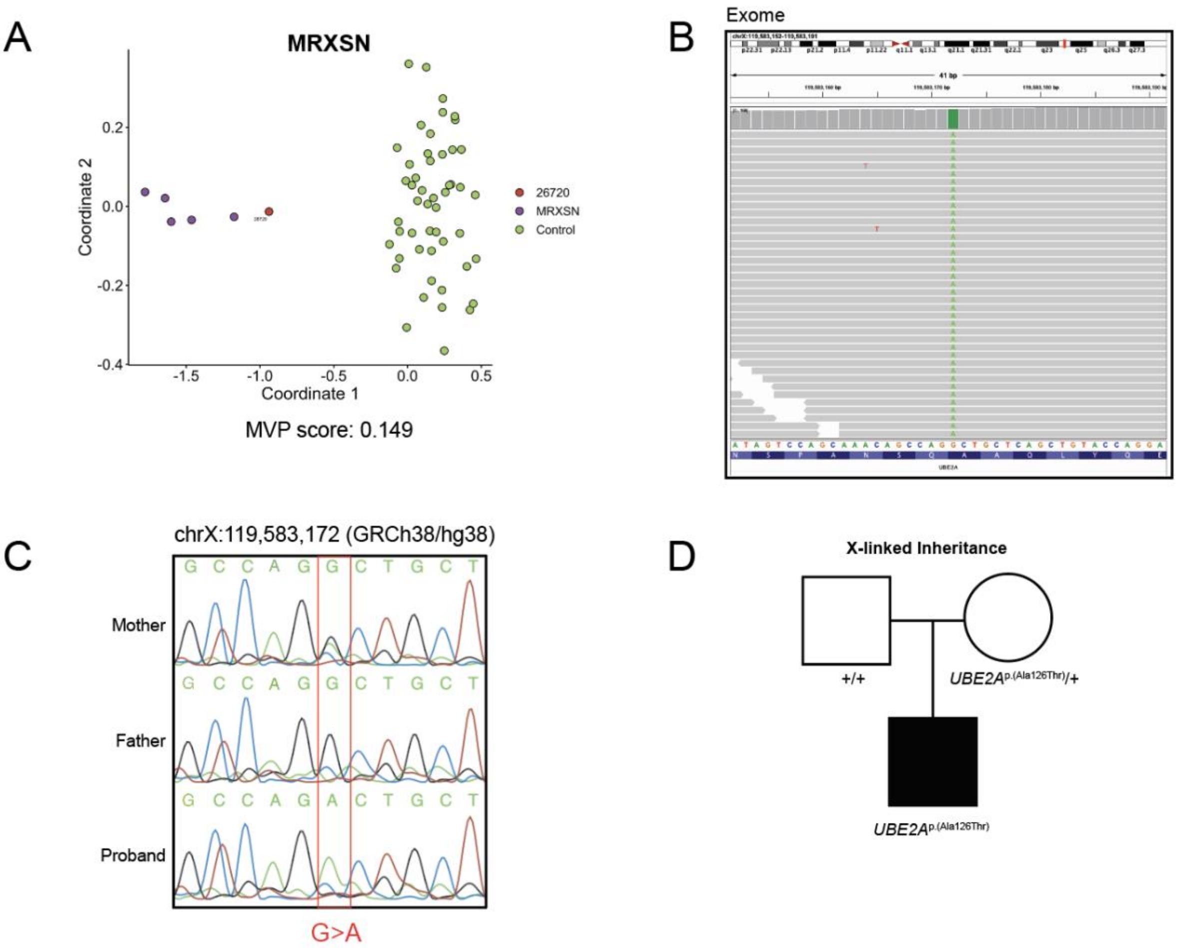

Supplementary Figure 22. Diagnosis of *UBE2A* enabled by episignature screening. **A**. MDS plot showing clustering of the proband with the MRXSN episignature. **B**. IGV screenshot of hemizygous variant of uncertain significance identified by exome sequencing. **C**. Sanger sequencing validation and segregation of variant, **D**. determined to X-linked and maternally inherited. Variant was determined to be pathogenic by clinical workup.

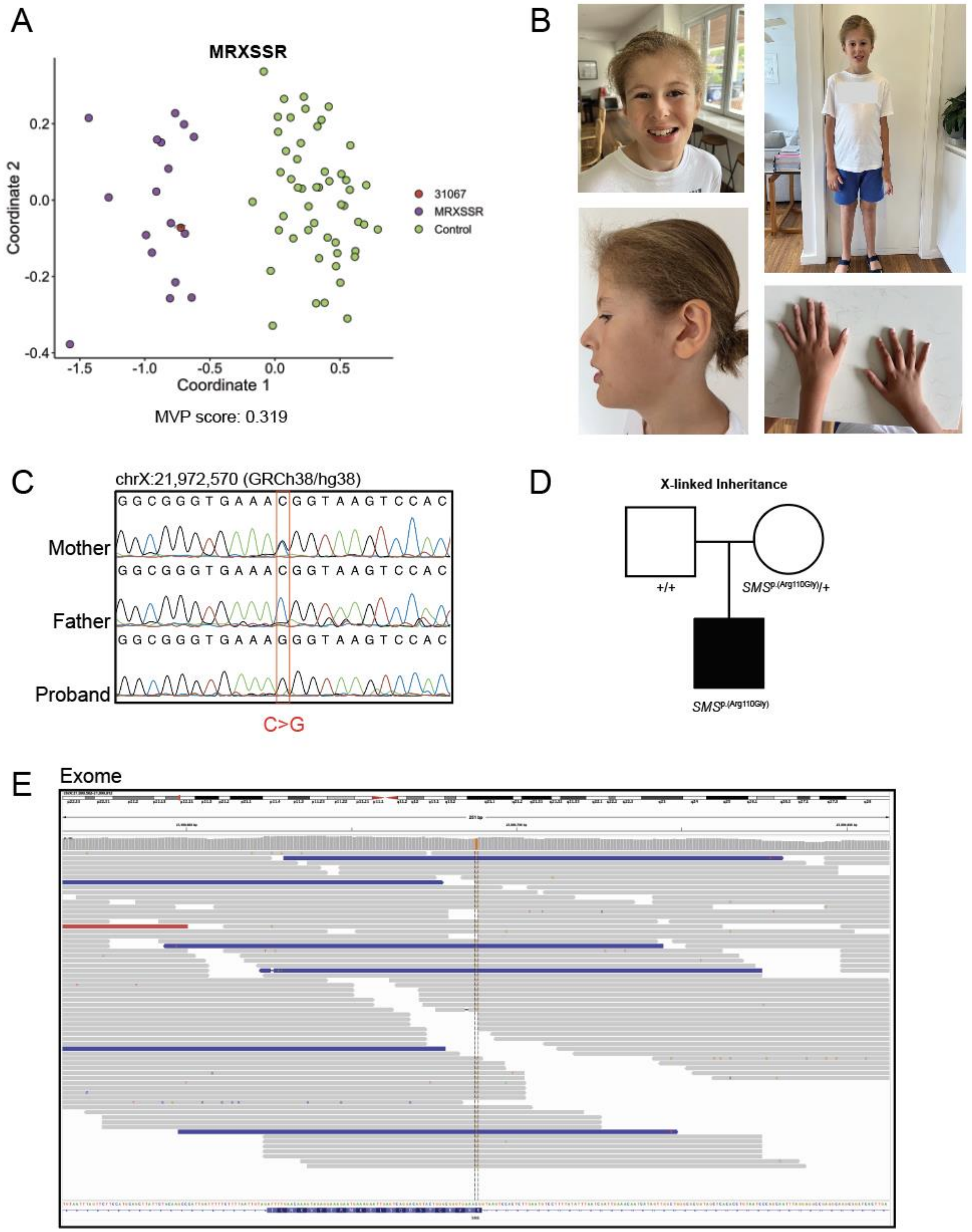
A
MRXSSR
Coordinate 2
Coordinate 1
31067
MRXSSR
Control
MVP score: 0.319
B
C
chrX:21,972,570 (GRCh38/hg38)
Mother
Father
Proband
C>G
D
X-linked Inheritance
+/+
SMSp.(Arg110Gly)/+
SMSp.(Arg110Gly)
E
Exome

Supplementary Figure 23. Diagnosis of *SMS* enabled by episignature screening. **A**. MDS plot showing clustering of the proband with the MRXSSR episignature. **B**. Photos of 31067 displaying dysmorphic features, including friable hair, thin upper lip, fine eyebrows, low anterior hairline, and long, thin fingers. **C**. Sanger sequencing validation and segregation of variant, **D**. determined to X-linked and maternally inherited. **E**. IGV screenshot of hemizygous variant identified by exome sequencing. Variant was determined to be pathogenic by clinical workup.

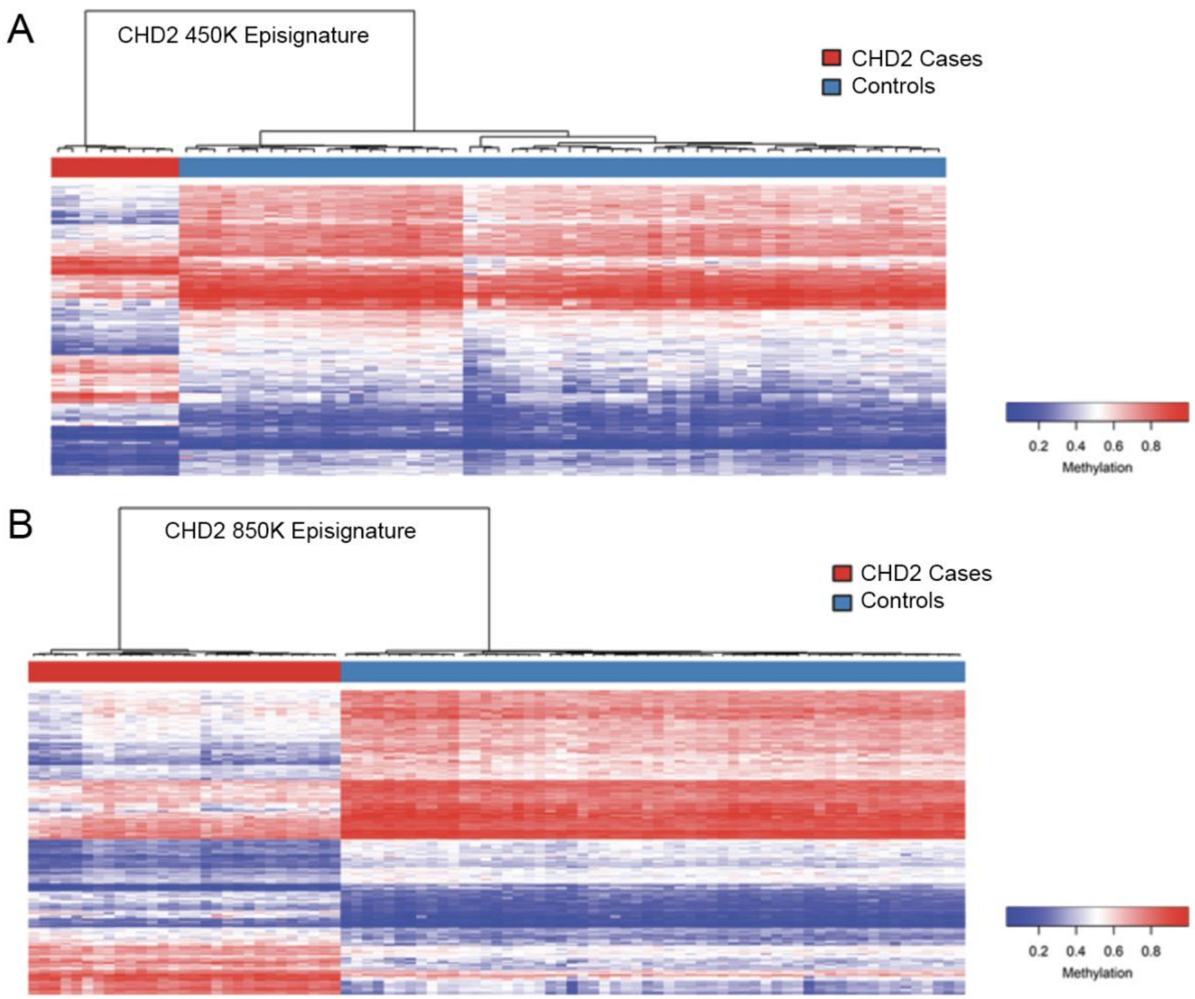

Supplementary Figure 24. Refinement of the *CHD2* Episignature on the 850K array. **A**. Heatmap and dendrogram showing clustering using the *CHD2* 450K episignature probes (n=200), derived using overlapping 450K/850K probes for n=9 individuals (n=2 450K and n=7 850K) with pathogenic variants in *CHD2* against n=54 controls. **B**. Heatmap and dendrogram showing clustering using the *CHD2* 850K episignature probes (n=200), derived using n=29 individuals with pathogenic variants in *CHD2* against n=58 controls.

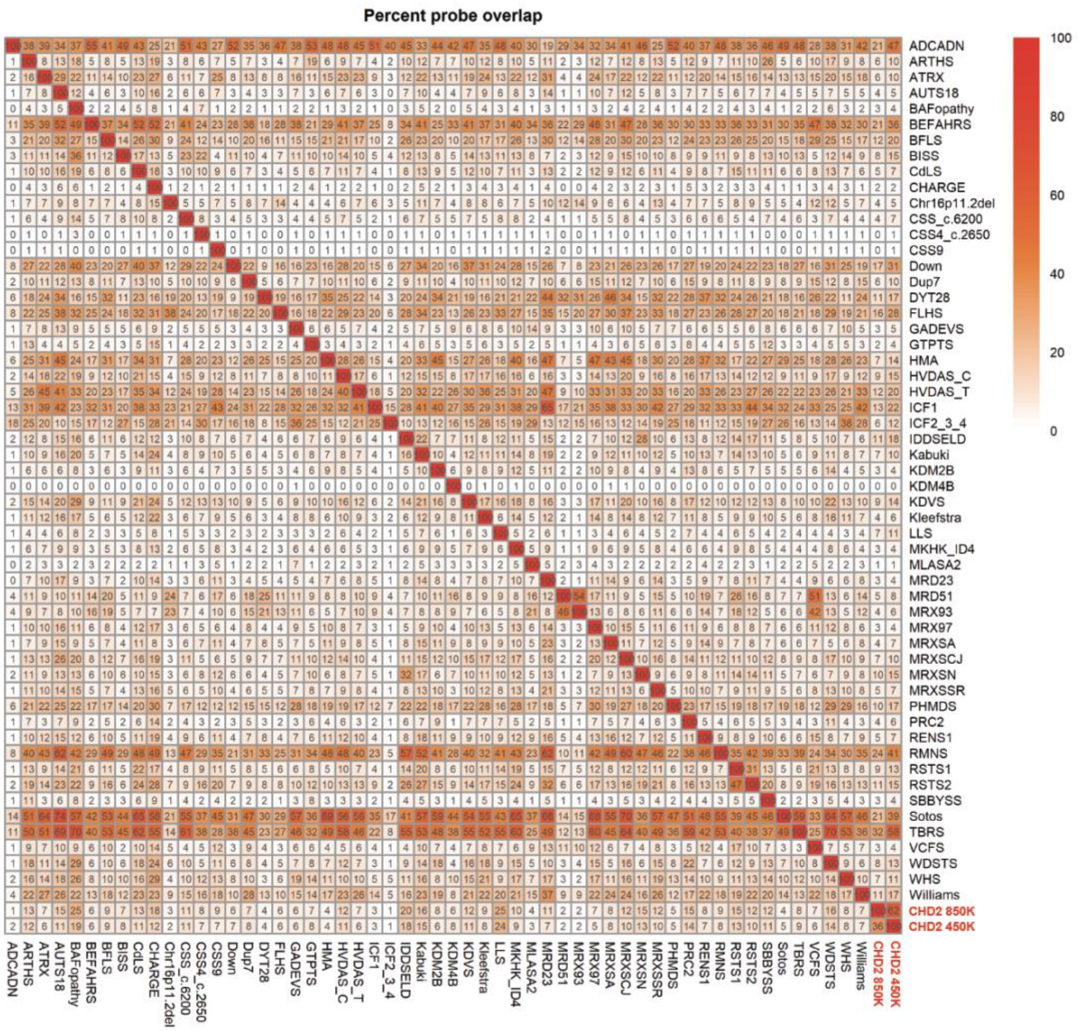

Supplementary Figure 25: Differentially methylated probes found to be shared between multiple cohorts. Percent of probes with an adjusted $p<0.01$ that are shared between each pair of cohorts (n=57 total cohorts) that was previously investigated[32]. For each pair, the colors indicate the percent of the bottom cohort's probes that are also found in the right cohort's probes. The CHD2 850K episignature shares some portion of probes with every other episignature represented except for the 3 episignatures with the smallest number of DMPs (<500 DMPs): KDM4B/*KDM4B* (MIM:619320), CSS9/*SOX11* (MIM:615866), and CSS4_c.2650/*SMARCA4*, a secondary signature for variants near SMARCA4:c.2650, which cluster separately from other CSS4/BAFopathy (MIM:614609) samples.

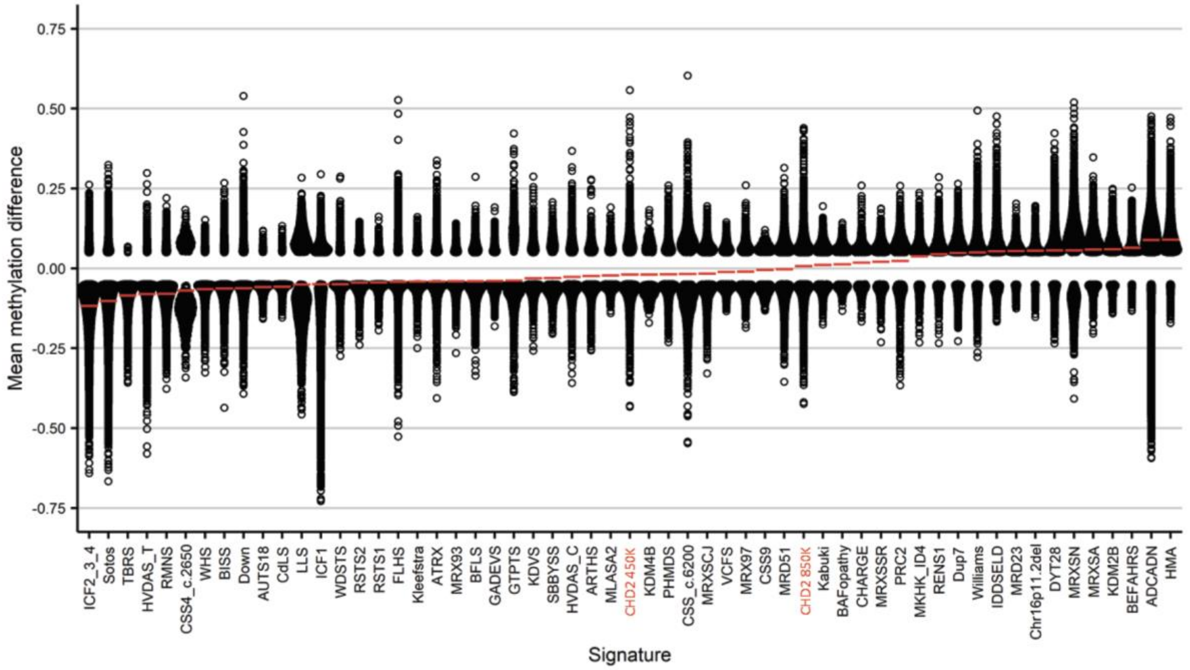

Supplementary Figure 26. Mean Methylation Difference Comparison Across Episignatures. Genome-wide DNA methylation profiles of the 450K and updated 850K CHD2 cohort and 55 other EpiSign^{TM} episignatures previously investigated for functional correlational analysis[32]. Global methylation differences of all differentially methylated probes (false discovery rate<0.05) for each cohort, sorted by mean methylation. Each circle represents 1 probe. Red lines indicate mean methylation. The x-axis represents 1 of the 57 episignatures and the y-axis is the mean methylation difference.

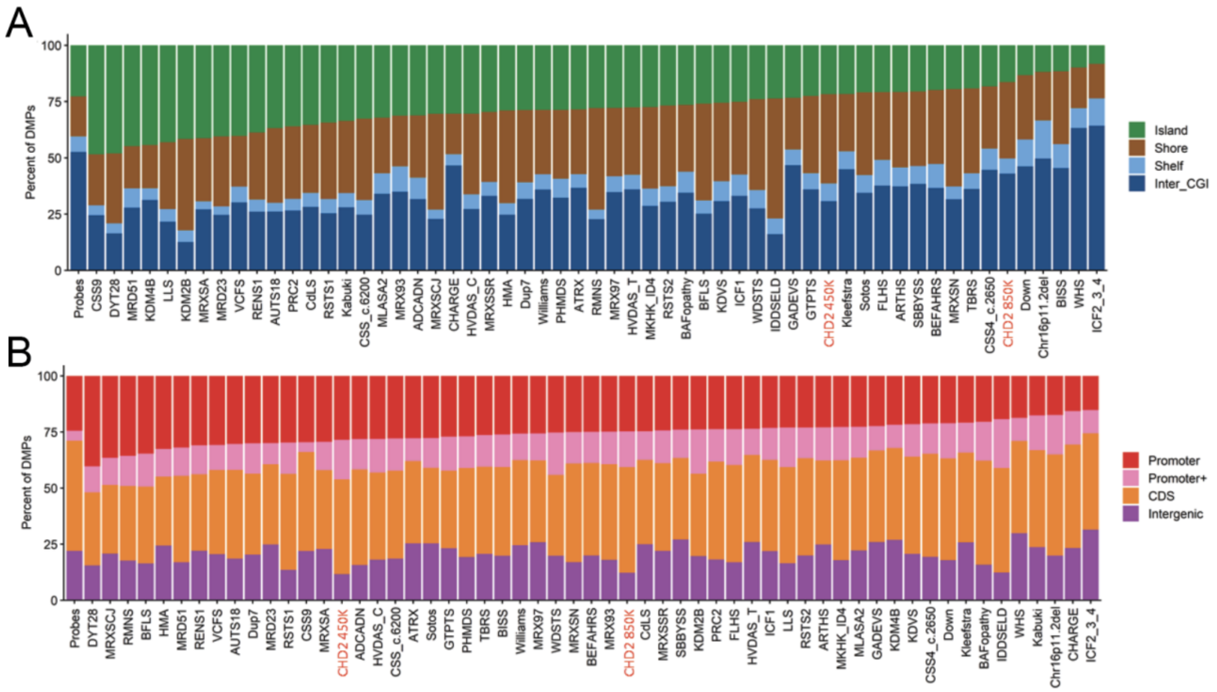

Supplementary Figure 27. Annotation of differentially methylated probes in 450K and 850K CHD2 episignatures and other EpiSign^{TM} episignatures previously published. Differentially methylated probes (DMPs) of CHD2 450K and 850K cohorts (red) and 56 other episignatures as previously investigated[32]. X-axis represents 1 of the 57 episignatures and the y-axis the percentage of DMPs. **A**. DMPs annotated in the context of CpG islands. Island, CpG islands; Shore, within 0 to 2 kb of a CpG island boundary; Shelf, within 2 to 4 kb of a CpG island boundary; inter-CGI, all other regions in the genome **B**. DMPs annotated in the context of genes. Promoter,0 to 1 kb upstream of the TSS; Promoter+, 1 to 5 kb upstream of the TSS; CDS, coding sequence; Intergenic, all other regions of the genome. The Probes column in panels A and B represents the background distribution determined in a previous study[33] of all array probes after initial filtering and used as input for DMP analysis.

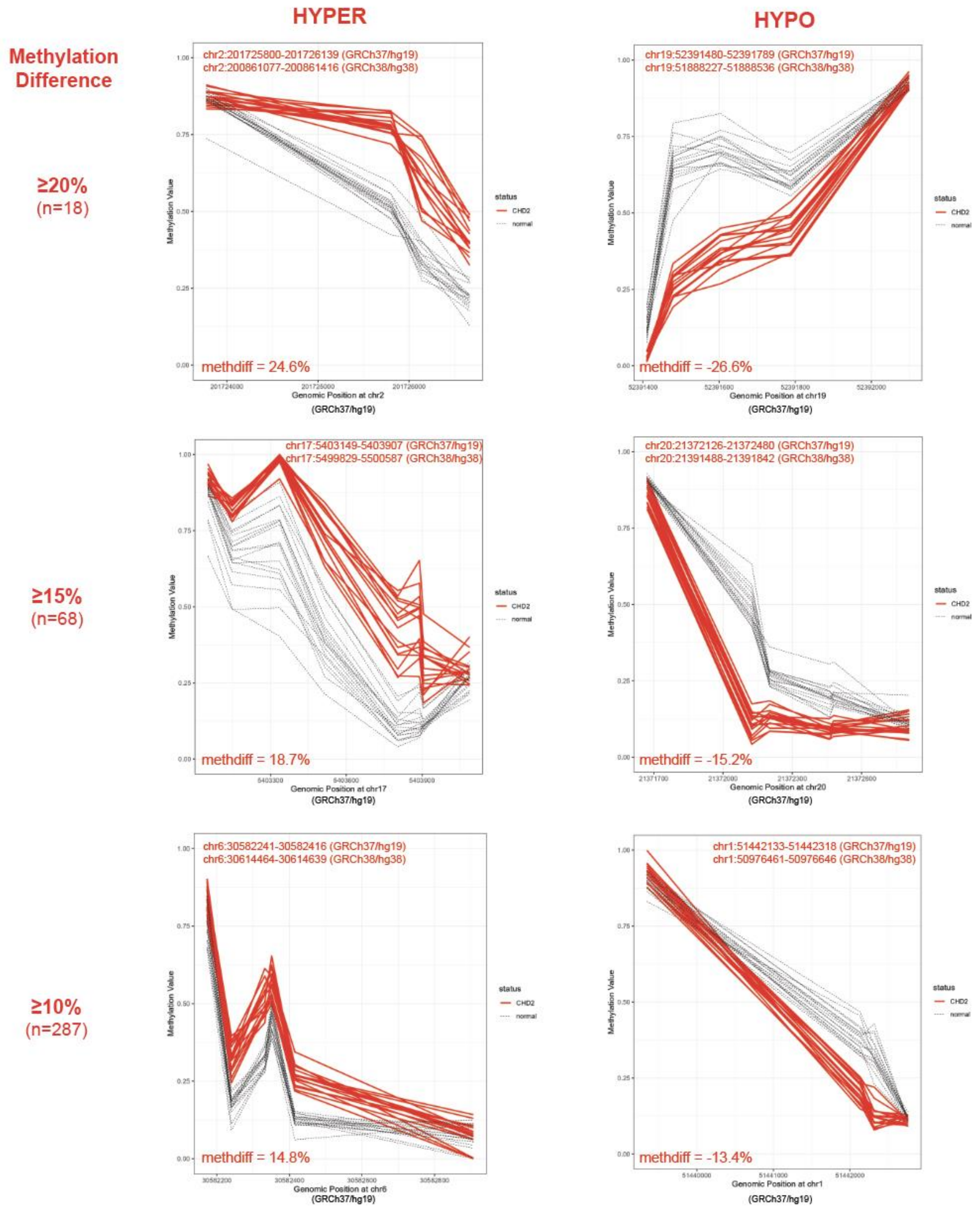

Supplementary Figure 28. Representative *CHD2* DMRs derived on the 850K array. DMRs were called from bumphunter and DMRcate for n=16 individuals with pathogenic variants in *CHD2* vs. n=18 unaffected controls. Final DMR coordinates were considered the overlapping regions of at least 50bp of more. Representative hypermethylated DMR plots (GRCh37/hg19) are shown on

the left, and hypomethylated DMR plots are shown on the right. The methylation differences were considered the average between the two callers and are shown in the DMR plots. The number of DMRs called at various methylation differences are noted to the left of the plots. A total of 712 DMRs were called with at least a 5% methylation difference between *CHD2* and controls.

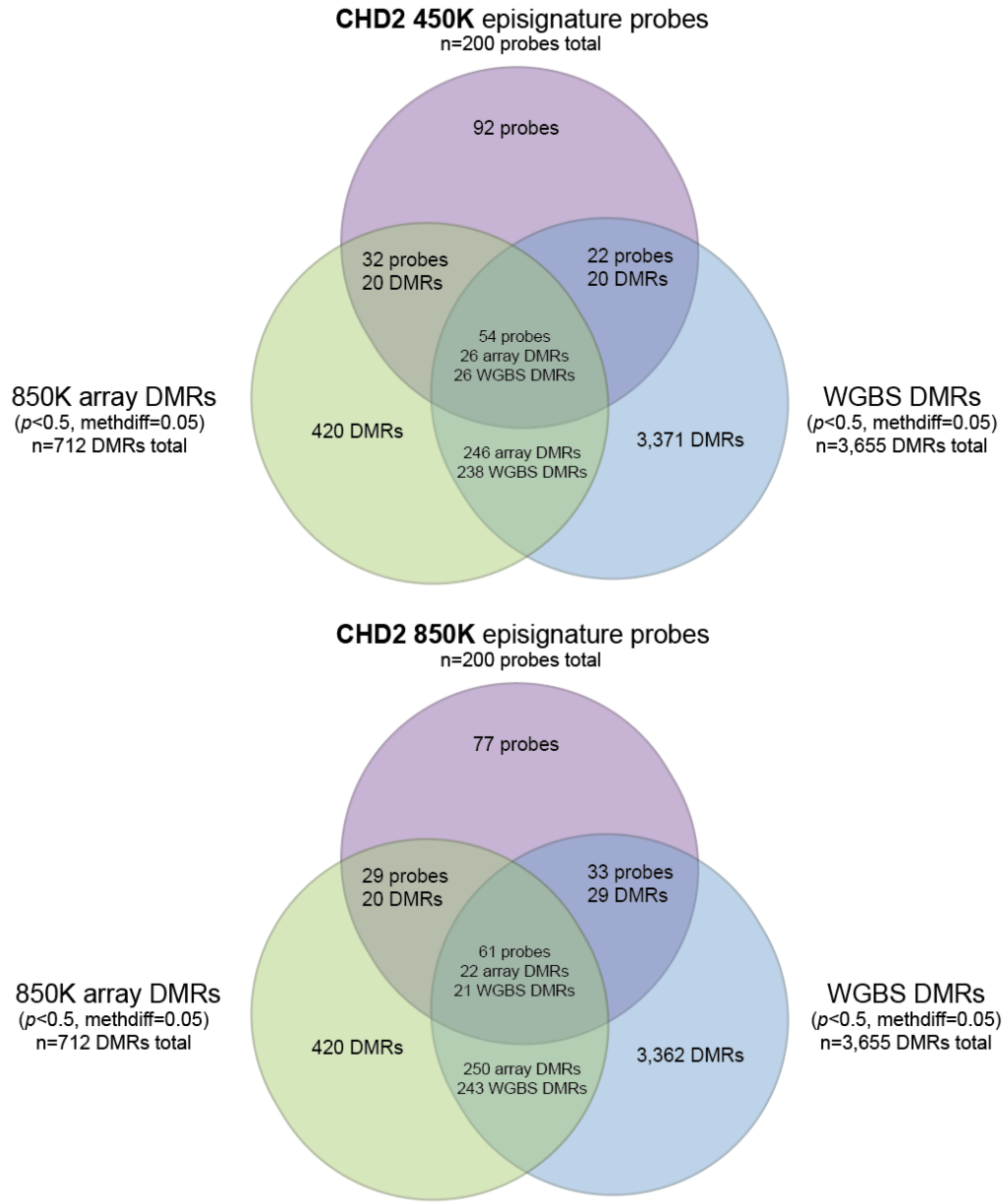

Supplementary Figure 29. Overlap of *CHD2* episignature probes with DMRs called from the 850K array and WGBS. Venn diagrams showing overlap of 450K CHD2 episignature (n=200 probes, top purple) and 850K (n=200 probes, bottom purple) with 850K array DMRs (left green) and WGBS DMRs (right blue). See methods for a detailed description of how episignatures and DMRs were derived.

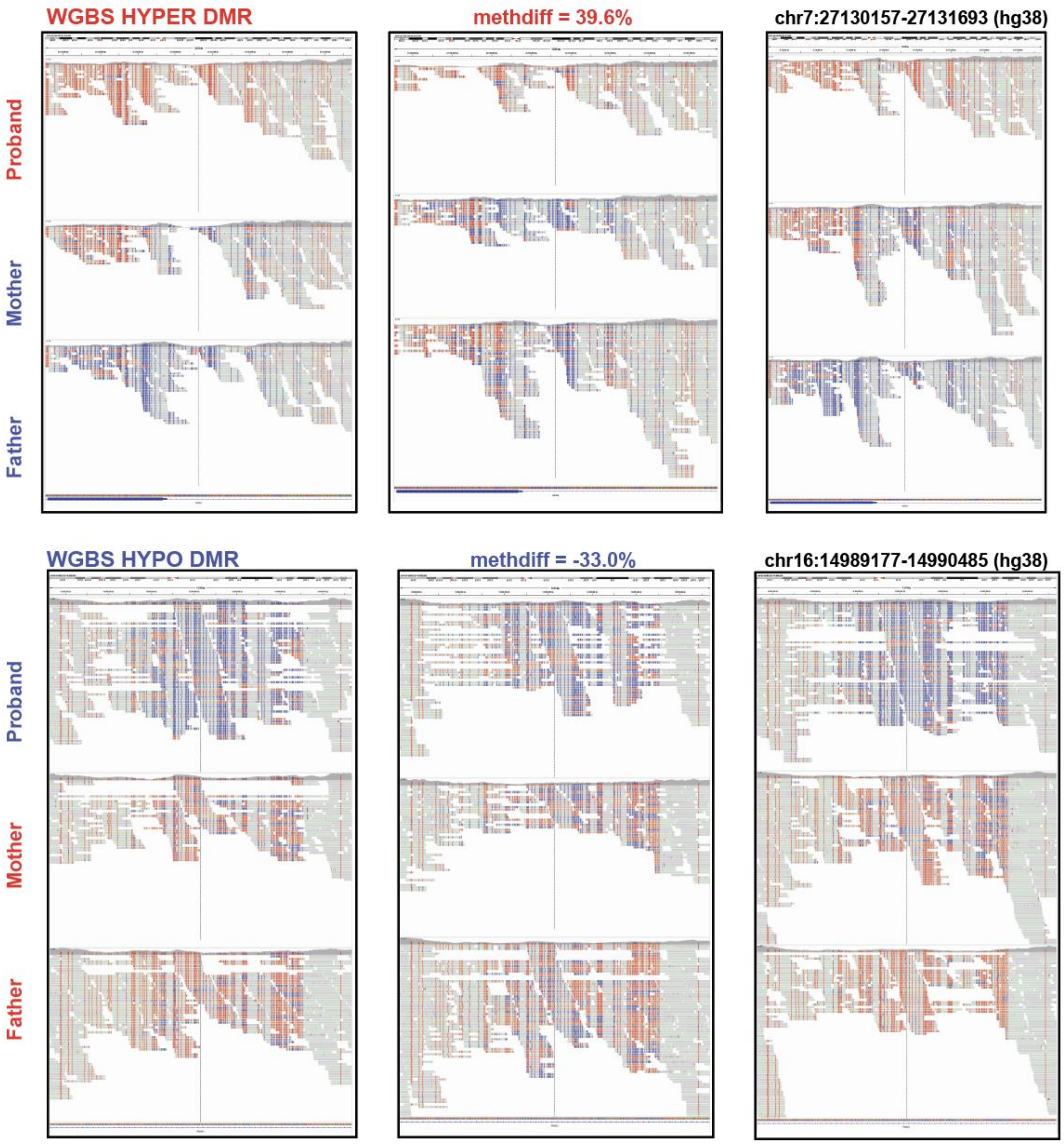

Supplementary Figure 30. Representative *CHD2* DMRs called from WGBS. DMRs were called for n=4 individuals with pathogenic variants in *CHD2* vs. n=6 unaffected parents requiring overlap from both DSS and DMRcate tools. Two representative DMRs (1 hyper, 1 hypo) are shown in IGV for n=3 trios. The proband (top track) is clearly differentially methylated compared to both parents (middle and bottom tracks). The center of the plot is marked by a dotted black line.

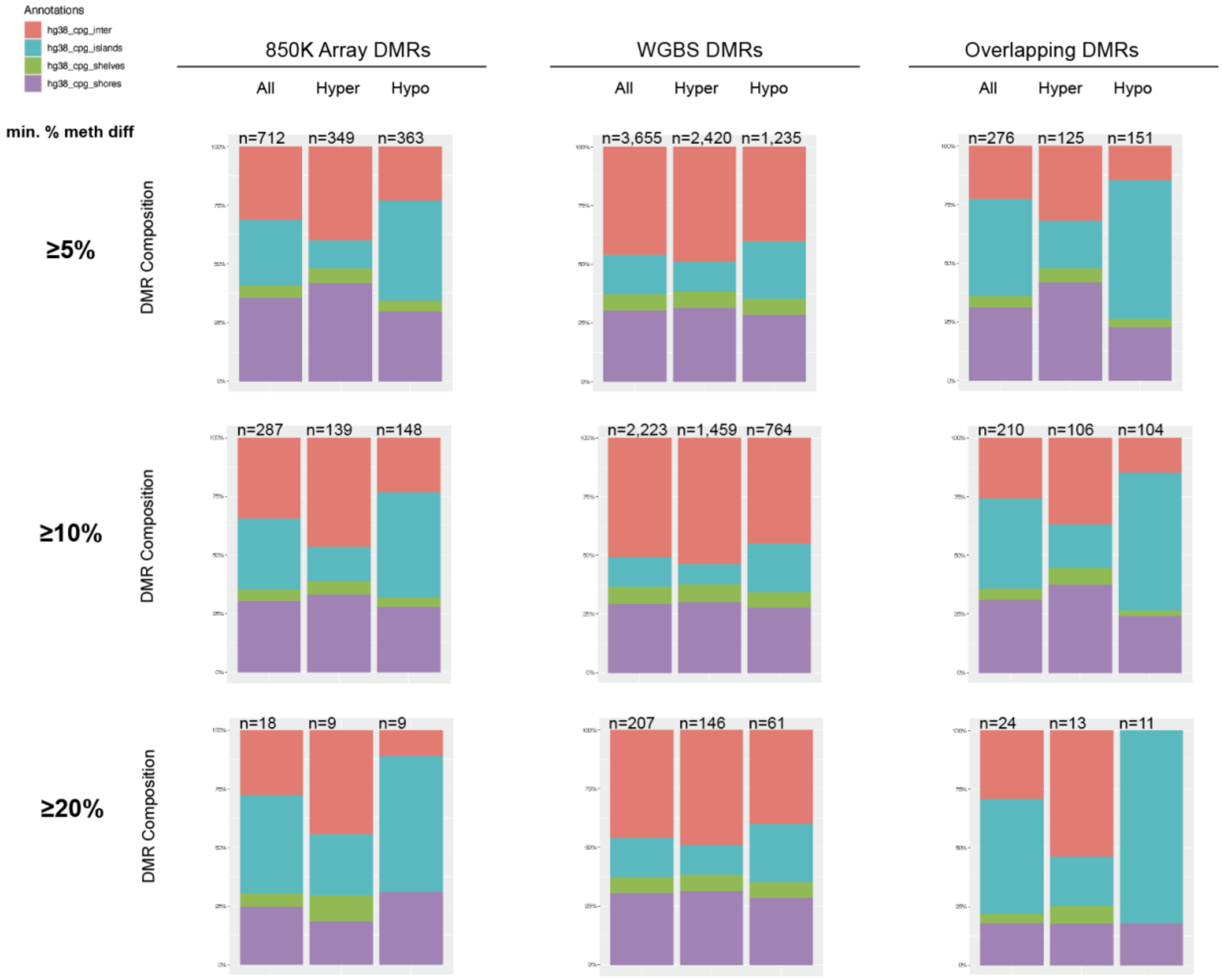

Supplementary Figure 31. Functional annotation of *CHD2* DMRs with CpG descriptions. DMRs were called for n=16 *CHD2* vs. n=18 unaffected controls using bumphunter and DMRcate for the 850K array and n=4 *CHD2* vs. n=6 unaffected controls using DSS and DMRcate for WGBS (Methods). DMRs were considered significant if $p<0.05$ and the percent methylation difference (% meth diff) was greater than or equal to the cutoffs shown (5%, 10%, 20%). DMR composition by CpG descriptors was calculated as a fraction of the length of each DMR to ensure that a DMR overlapping with multiple descriptors was only counted once. CpG islands are areas of the genome with a high density of CpGs. CpG shores are located within 2Kb on either side of CpG

islands. CpG shelves are located within 2Kb of CpG shores, and interCpG islands (interCGI) covers the rest of the genome.

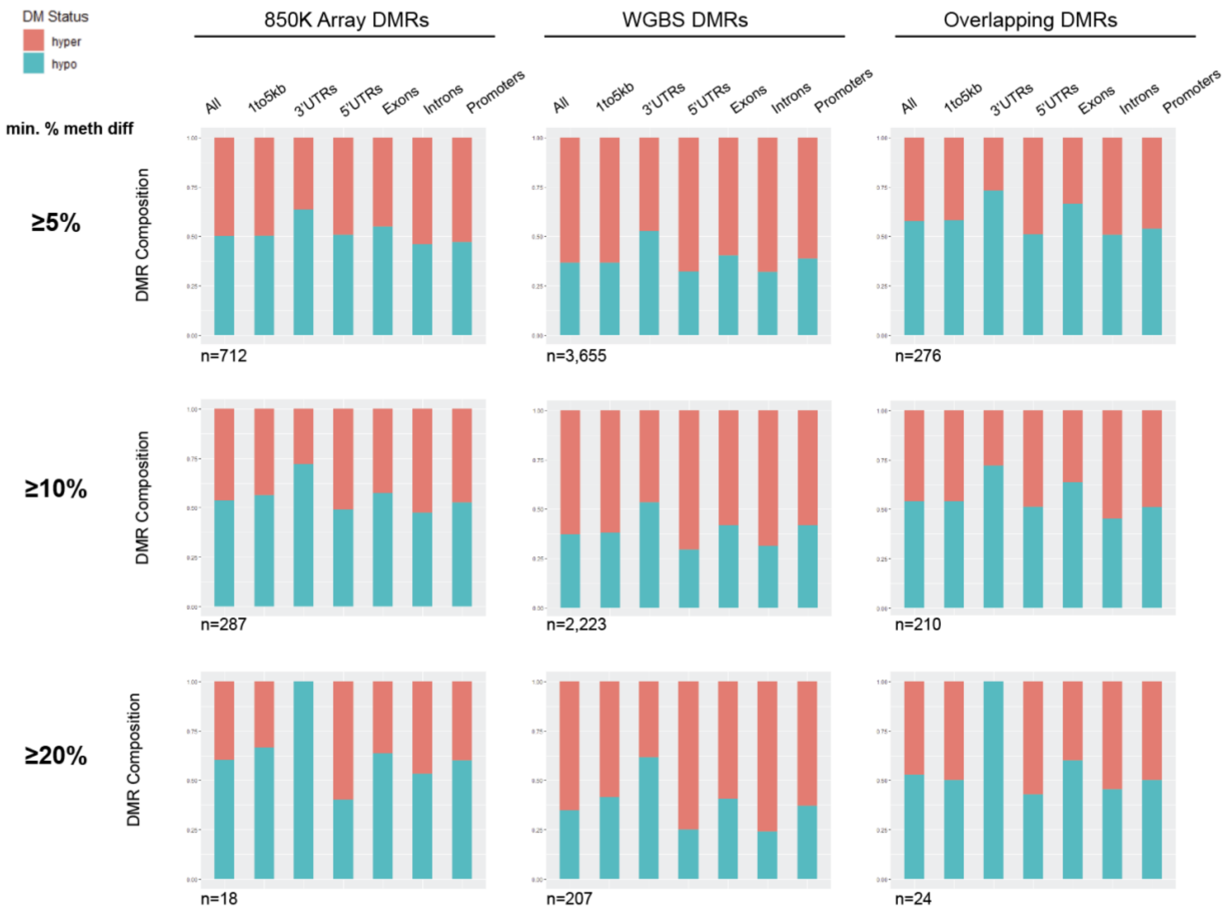

Supplementary Figure 32. Functional annotation of *CHD2* DMRs by gene region. DMRs were called for n=16 *CHD2* vs. n=18 unaffected controls using bumphunter and DMRcate for the 850K array and n=4 *CHD2* vs. n=6 unaffected controls using DSS and DMRcate for WGBS (Methods). DMRs were considered significant if $p<0.05$ and the percent methylation difference (% meth diff) was greater than or equal to the cutoffs shown (5%, 10%, 20%). DMR composition by gene region includes 1-5Kb upstream of the TSS (1to5kb), the promoters (<1Kb upstream the TSS), the 5'UTRs, exons, introns, and the 3'UTRs. Representation as a fraction across hypermethylated DMRs are shown in pink and hypomethylated DMRs are shown in blue.

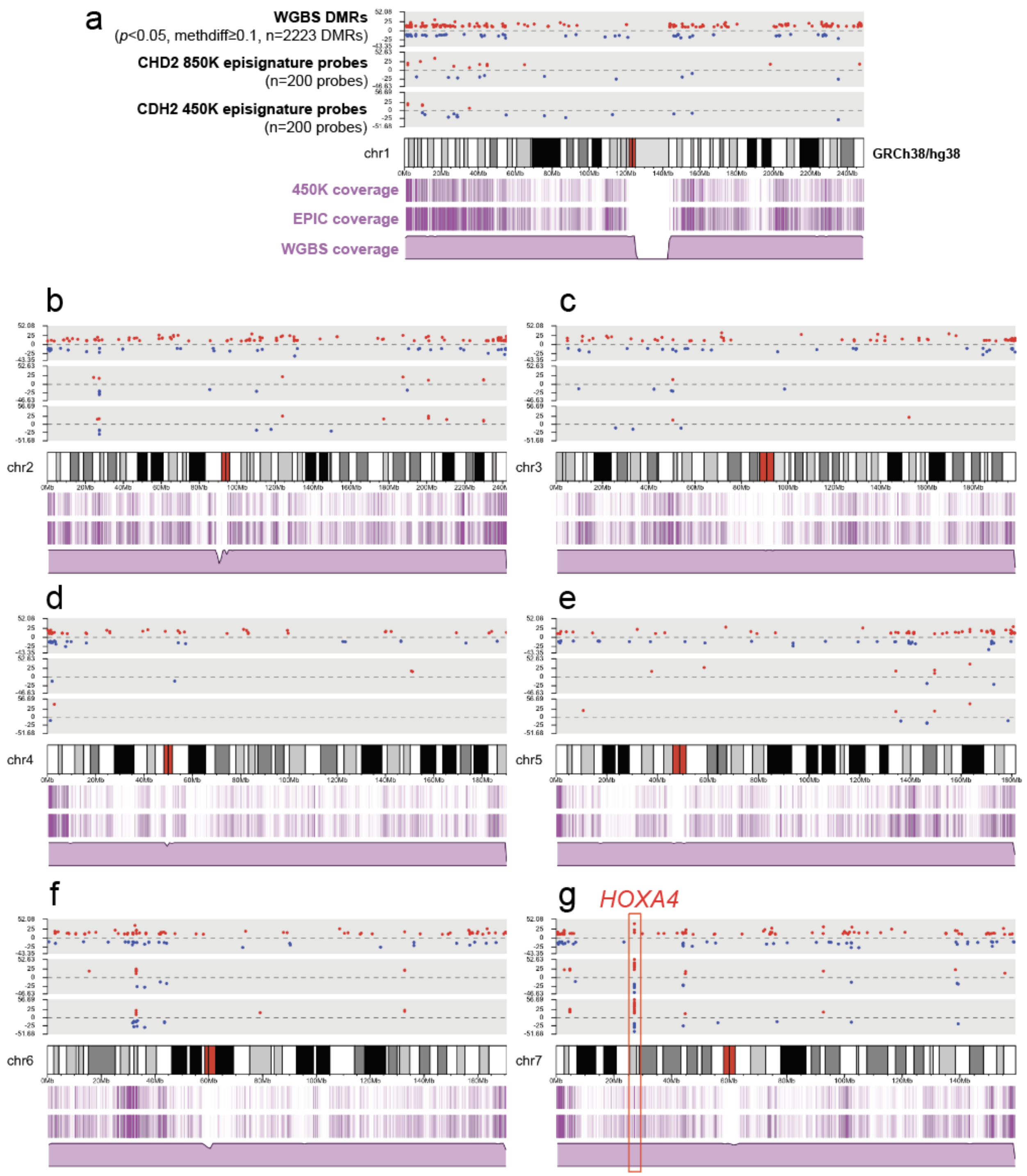

a
WGBS DMRs
(p<0.05, methdiff≥0.1, n=2223 DMRs)
CHD2 850K episignature probes
(n=200 probes)
CDH2 450K episignature probes
(n=200 probes)
chr1
GRCh38/hg38
450K coverage
EPIC coverage
WGBS coverage
b
c
d
e
f
g
HOXA4
chr2
chr3
chr4
chr5
chr6
chr7

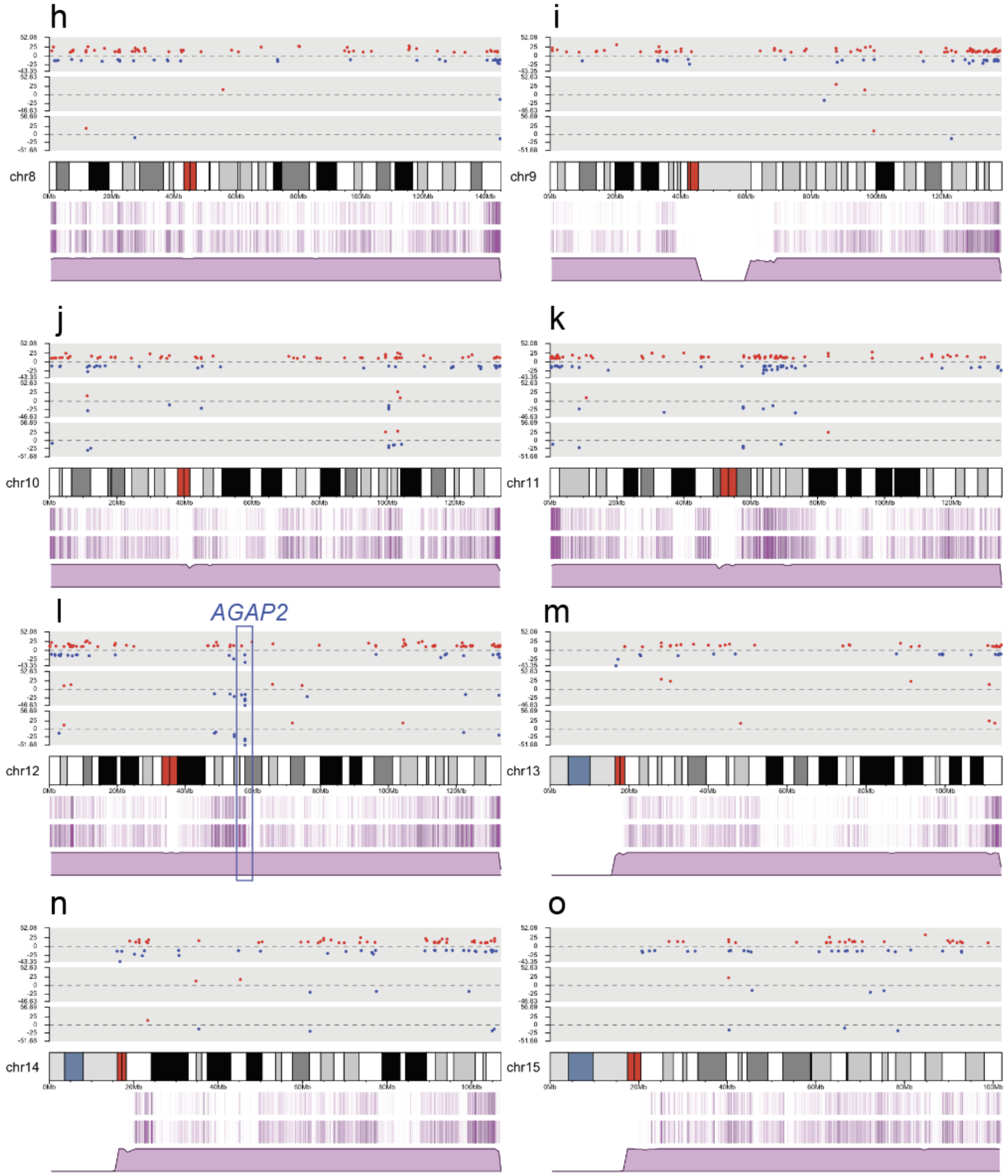
h
i
j
k
l
AGAP2
m
n
o
chr8
chr9
chr10
chr11
chr12
chr13
chr14
chr15

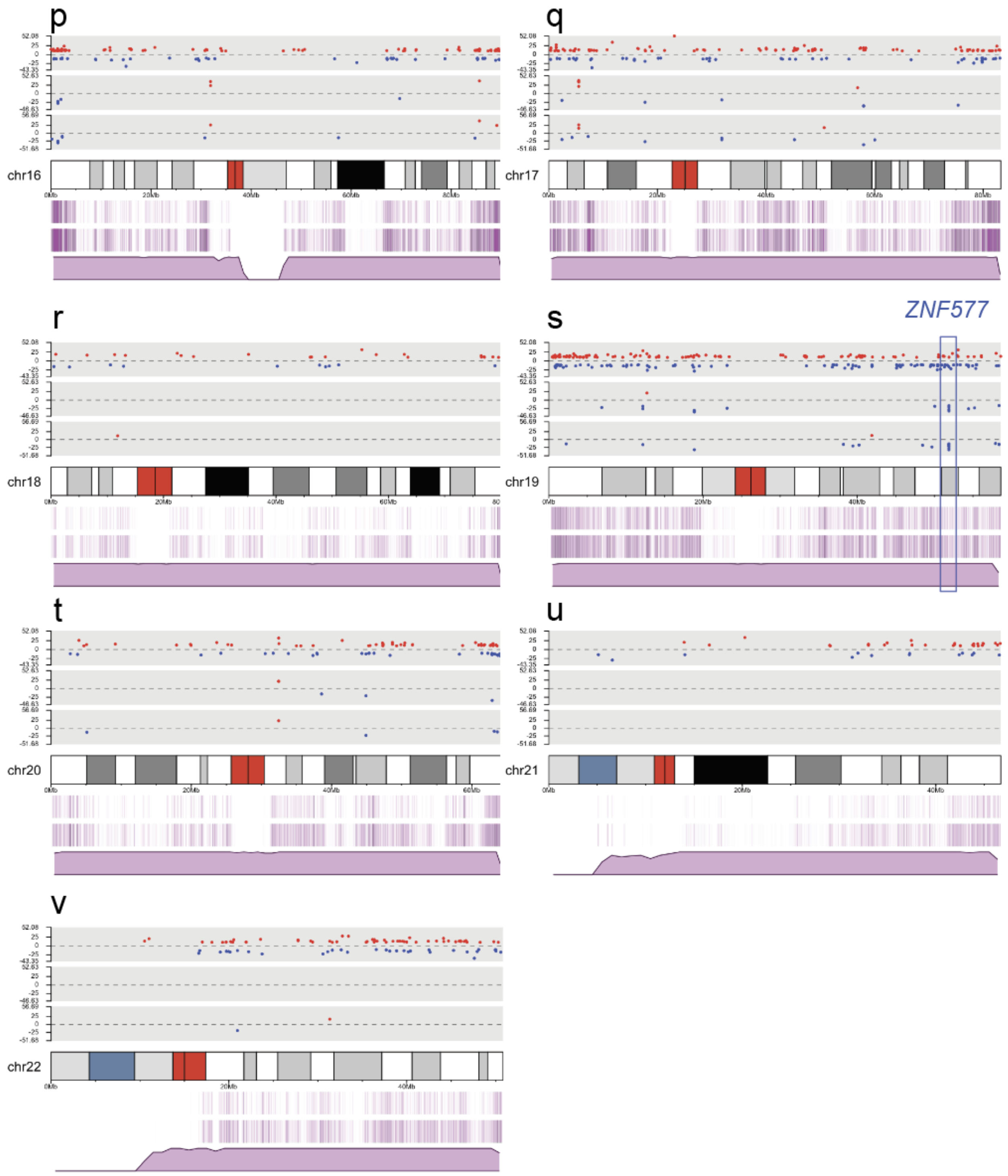

Supplementary Figure 33. Linear karyotype plots representing *CHD2* episignature probes overlapping with DMRs called from n=4 *CHD2* vs. n=6 unaffected controls using WGBS. Individual karyotype tracks for chr1-22 (**a-v**) where three grey tracks (upper panel above

chromosome) depict individual red (hyper) or blue (hypo) dots for WGBS DMRs (upper), CHD2 850K episignature probes (middle) and CHD2 450K episignature probes (lower). The scale denotes the methylation difference between *CHD2* relative to controls. Three purple tracks (lower panel below chromosome) depict the coverage for the 450K array probes (lines, upper), 850K array probes (lines, middle), and WGBS reads (distribution, lower). The coverage track for the WGBS is taken from a representative sample after inspecting the average coverage values across all the samples. Examples of where episignature probes form a cluster and overlap with a DMR are boxed in red (hyper) and blue (hypo) for *HOXA4*, *AGAP*2, and *ZNF577*.

Full List of Author Abbreviations

Christy W. LaFlamme	C.W.L.
Cassandra Rastin	C.R.
Soham Sengupta	S.S.
Helen E. Pennington	H.E.P.
Sophie J. Russ-Hall	S.J.R.
Amy L. Schneider	A.L.S.
Emily S. Bonkowski	E.S.B.
Edith P. Almanza Fuerte	E.P.A.
Talia J. Allan	T.J.A.
Miranda Perez-Galey Zalusky	M.P.Z.
Joy Goffena	J.G.
Sophia B. Gibson	S.B.G.
Denis M. Nyaga	D.M.N.
Nico Lieffering	N.L.
Malavika Hebbar	M.H.
Emily V. Walker	E.V.W.
Daniel Darnell	D.D.
Scott R. Olsen	S.R.O.
Pandurang Kolekar	P.K.
Mohamed Nahdir Djekidel	M.N.D.
Wojciech Rosikiewicz	W.R.
Haley McConkey	H.M.
Jennifer Kerkhof	J.K.

751	Michael A. Levy	M.A.L.
752	Raissa Relator	R.R.
753	Dorit Lev	D.L.
754	Tally Lerman-Sagie	T.L.S.
755	Kristen L. Park	K.L.P.
756	Marielle Alders	M.A.
757	Gerarda Cappuccio	G.C.
758	Nicolas Chatron	N.C.
759	Leigh Demain	L.D.
760	David Genevieve	D.G.
761	Gaetan Lesca	G.L.
762	Tony Roscioli	T.R.
763	Damien Sanlaville	D.S.
764	Matthew L. Tedder	M.L.T.
765	Sachin Gupta	S.G.
766	Elizabeth A. Jones	E.A.J.
767	Monika Weisz-Hubshman	M.W.H.
768	Shamika Ketkar	S.K.
769	Hongzheng Dai	H.D.
770	Kim C. Worley	K.C.W.
771	Jill A. Rosenfeld	J.A.R.
772	Hsiao-Tuan Chao	H.T.C.
773	Undiagnosed Diseases Network	UDN

Geoffrey Neale	G.N.
Gemma L. Carvill	G.L.C.
University of Washington Center for Rare Disease Research	UW-CRDR
Zhaoming Wang	Z.W.
Samuel F. Berkovic	S.F.B.
Lynette G. Sadleir	L.G.S.
Danny E. Miller	D.E.M.
Ingrid E. Scheffer	I.E.S.
Bekim Sadikovic	B.S.
Heather C. Mefford	H.C.M.

Full List of Consortia Members for UW-CRDR and UDN

University of Washington Center for Rare Disease Research (UW-CRDR)

Chia-Lin Wei[1], Michael J. Bamshad[1,2] and Evan E. Eichler[1,3]

Kailyn Anderson[1], Peter Anderson[1], Tamara J. Bacus[1], Sabrina Best[1], Elizabeth E. Blue[1], Katherine Brower[1], Kati J. Buckingham[1], Brianne Carroll[1], Silvia Casadei[1], Jessica X. Chong[1], Nikhita Damaraju[1], Colleen P. Davis[1], Christian D. Frazar[1], Sophia Gibson[1], Joy Goffena[1], William W. Gordon[1], Jonas A. Gustafson[1], William T. Harvey[1], Martha Horike-Pyne[1], Jameson R. Hurless[1], Caitlin Jacques[1], Gail P. Jarvik[1], Eric Johanson[1], J. Thomas Kolar[1], Xiaomeng Liu[1], Colby T. Marvin[1], Sean McGee[1], Holli Meyers[1], Heather Mefford[4], Danny E. Miller[1,2], Patrick M. Nielsen[1], Karynne Patterson[1], Aparna Radhakrishnan[1], Matthew A. Richardson[1], Erica L. Ryke[1], Aliya Sarkytbayeva[1], Tristan Shaffer[1], Kathryn M. Shively[1], Olivia M. Sommers[1], Sophie H.R. Storz[1], Joshua D. Smith[1], Lea M. Starita[1], Monica Tackett[1], Sydney A. Ward[1], Jeffrey M. Weiss[1], Qian Yi[1], and Miranda P.G. Zalusky[1].

[1]University of Washington

[2]Seattle Children's Hospital

[3]Howard Hughes Medical Institute

[4]St. Jude Children's Research Hospital

**Members of the Undiagnosed Diseases Network (Version: 6.3.2024)**		
**Full Name**	**Affiliation**	**Email**
Aaron Quinlan	University of Utah	aquinlan@genetics.utah.edu
Adeline Vanderver	CHOP	vandervera@chop.edu
Adriana Rebelo	Miami	arebelo@med.miami.edu
Aimee Allworth	PNW	allwoa@uw.edu
Alan H. Beggs	Harvard	beggs@enders.tch.harvard.edu
Alden Huang	UCLA	AYHuang@mednet.ucla.edu
Alex Paul	WUSTL Clinical	alex.paul@wustl.edu
Ali Al-Beshri	UAB	asabeshri@uabmc.edu
Allyn McConkie-Rosell	Duke	allyn.mcconkie@duke.edu
Alyssa A. Tran	BCM Clinical	alyssat@bcm.edu
Andrea Gropman	NIH UDP	agropman@childrensnational.org
Andrew B. Crouse	UAB DMCC	acrouse@uab.edu
Andrew Stergachis	PNW	absterga@uw.edu
Anita Beck	PNW	aebeck@uw.edu
Anna Hurst	UAB	acehurst@uab.edu
Anna Raper	CHOP/UPenn	rapera@pennmedicine.upenn.edu

Anne Hing	PNW	anne.hing@seattlechildrens.org
Arjun Tarakad	BCM Clinical	tarakad@bcm.edu
Ashley Andrews	University of Utah	ashley.andrews@hsc.utah.edu
Ashley McMinn	Vanderbilt	ashley.mcminn@vumc.org
Ashok Balasubramanyam	BCM Clinical	ashokb@bcm.edu
AudreyStephannie C. Maghiro	Harvard DMCC	audreystephannie_maghiro@hms.harvard.edu
Barbara N. Pusey Swerdzewski	NIH UDP	barbara.pusey@nih.gov
Ben Afzali	NIH UDP, NHGRI	ben.afzali@nih.gov
Ben Solomon	NIH UDP, NHGRI	solomonb@mail.nih.gov
Beth A. Martin	Stanford	martinb@stanford.edu
Breanna Mitchell	Mayo Clinic	Mitchell.Breanna@mayo.edu
Brendan C. Lanpher	Mayo Clinic	lanpher.brendan@mayo.edu
Brendan H. Lee	BCM Clinical	blee@bcm.edu
Brent L. Fogel	UCLA	bfogel@ucla.edu
Brett H. Graham	IU	bregraha@iu.edu
Brian Corner	Vanderbilt	brian.corner@vumc.org
Bruce Korf	UAB	bkorf@uab.uabmc.edu
Calum A. MacRae	Harvard	camacrae@bics.bwh.harvard.edu

Camilo Toro	NIH UDP	toroc@mail.nih.gov
Cara Skraban	CHOP	skrabanc@chop.edu
Carlos A. Bacino	BCM Clinical	cbacino@bcm.edu
Carson A. Smith	Miami	carsonsmith@med.miami.edu
Cecilia Esteves	Harvard DMCC	cecilia_esteves@hms.harvard.edu
Changrui Xiao	UCI/CHOC	changrx@hs.uci.edu
Chloe M. Reuter	Stanford	creuter@stanfordhealthcare.org
Christina Lam	PNW	ctlam2@uw.edu
Christine M. Eng	BCM Sequencing	ceng@bcm.edu
Claire Henchcliffe	UCI/CHOC	chenchcl@hs.uci.edu
Colleen E. Wahl	NIH UDP	colleen.wahl@nih.gov
Corrine K. Welt	University of Utah	cwelt@u2m2.utah.edu
Cynthia J. Tifft	NIH UDP, NHGRI	ctifft@nih.gov
Dana Kiley	WUSTL Clinical	dana.kiley@wustl.edu
Daniel Doherty	PNW	ddoher@uw.edu
Daniel J. Rader	CHOP/UPenn	rader@pennmedicine.upenn.edu
Daniel Wegner	WUSTL Clinical	danieljwegner@wustl.edu
Danny Miller	PNW	Danny.Miller@seattlechildrens.org
Daryl A. Scott	BCM Clinical	dscott@bcm.edu

Dave Viskochil	University of Utah	dave.viskochil@hsc.utah.edu
David A. Sweetser	Harvard	dsweetser@partners.org
David R. Adams	NIH UDP, NHGRI	david.adams@nih.gov
Dawn Earl	PNW	dawn.earl@seattlechildrens.org
Deborah Barbouth	Miami	dbarbouth@miami.edu
Deborah Krakow	UCLA	dkrakow@mednet.ucla.edu
Deepak A. Rao	Harvard	darao@bwh.harvard.edu
Devin Oglesbee	Mayo Clinic	oglesbee.devin@mayo.edu
Devon Bonner	Stanford	devonbonner@stanfordhealthcare.org
Donna Novacic	NIH UDP	donna.novacic@nih.gov
Dustin Baldridge	WUSTL MOSC	dbaldri@wustl.edu
Edward Behrens	CHOP	behrens@chop.edu
Edwin K. Silverman	Harvard	ed.silverman@channing.harvard.edu
Elaine Seto	BCM Clinical	esseto@bcm.edu
Elijah Kravets	Stanford	ekravets@stanford.edu
Elizabeth A. Burke	NIH UDP, NHGRI	elizabeth.burke2@nih.gov
Elizabeth Blue	PNW	em27@uw.edu
Elizabeth L. Fieg	Harvard	efieg@bwh.harvard.edu
Elizabeth Rosenthal	PNW	erosen@uw.edu

Ellen F. Macnamara	NIH UDP	ellen.macnamara@nih.gov
Elsa Balton	PNW	ebalton@medicine.washington.edu
Emilie D. Douine	UCLA	edouine@mednet.ucla.edu
Emily Glanton	Harvard DMCC	Emily_Glanton@hms.harvard.edu
Emily Shelkowitz	PNW	
Eric Allenspach	PNW	eric.allenspach@seattlechildrens.org
Eric Klee	Mayo Clinic	klee.eric@mayo.edu
Eric Vilain	UCI/CHOC	evilain@hs.uci.edu
Erin Baldwin	University of Utah	erin.baldwin@hsc.utah.edu
Erin Conboy	IU	econboy@iu.edu
Erin McRoy	WUSTL Clinical	e.hediger@wustl.edu
Esteban C. Dell'Angelica	UCLA	edellangelica@mednet.ucla.edu
Euan A. Ashley	Stanford DMCC	Euan@stanford.edu
F. Sessions Cole	WUSTL DMCC	fcole@wustl.edu
Filippo Pinto e Vairo	Mayo Clinic	vairo.filippo@mayo.edu
Frances High	Harvard	fhigh@partners.org
Francesco Vetrini	IU	fvetrini@iu.edu
Francis Rossignol	NIH UDP, NHGRI	francis.rossignol@nih.gov
Fuki M. Hisama	PNW	fmh2@uw.edu

Gabor Marth	University of Utah DMCC	gmarth@genetics.utah.edu
Gail P. Jarvik	PNW	pair@uw.edu
Gary D. Clark	BCM Clinical	gdclark@texaschildrens.org
George Carvalho	UCLA	GCarvalhoNeto@mednet.ucla.edu
Gerard T. Berry	Harvard	gerard.berry@childrens.harvard.edu
Ghayda Mirzaa	PNW	gmirzaa@uw.edu
Gill Bejerano	Stanford	bejerano@stanford.edu
Giorgio Sirugo	CHOP/UPenn	Giorgio.Sirugo@pennmedicine.upenn.edu
Gonench Kilich	CHOP	kilichg@chop.edu
Guney Bademci	Miami	g.bademci@miami.edu
Heidi Wood	NIH UDP, NHGRI	heidi.wood@nih.gov
Herman Taylor	Morehouse DMCC	htaylor@msm.edu
Holly K. Tabor	Stanford	hktabor@stanford.edu
Hongzheng Dai	BCM Clinical	Hongzheng.Dai@bcm.edu
Hsiao-Tuan Chao	BCM Clinical	hc140077@bcm.edu
Hugo J. Bellen	BCM MOSC	hbellen@bcm.edu
Ian Glass	PNW	ianglass@uw.edu
Ian R. Lanza	Mayo Clinic	lanza.ian@mayo.edu
Ingrid A. Holm	Harvard	ingrid.holm@childrens.harvard.edu

Isaac S. Kohane	Harvard DMCC	isaac_kohane@hms.harvard.edu
Ivan Chinn	BCM Clinical	Ivan.Chinn@bcm.edu
J. Carl Pallais	Harvard	Juan.Pallais@mgh.harvard.edu
Jacinda B. Sampson	Stanford	jacindas@stanford.edu
James P. Orengo	BCM Clinical	james.orengo@bcm.edu
Jason Hom	Stanford	jasonhom@stanford.edu
Jennefer N. Kohler	Stanford	jkohler@stanfordhealthcare.org
Jennifer E. Posey	BCM Clinical	Jennifer.Posey@bcm.edu
Jennifer Wambach	WUSTL Clinical	wambachj@wustl.edu
Jessica Douglas	Harvard	Jessica.Douglas@childrens.harvard.edu
Jiayu Fu	NIH UDP, NHGRI	fuj6@mail.nih.gov
Jill A. Rosenfeld	BCM Clinical	mokry@bcm.edu
Jimann Shin	WUSTL MOSC	shinji@wustl.edu
Jimmy Bennett	PNW	jtbenn@uw.edu
Joan M. Stoler	Harvard	joan.stoler@childrens.harvard.edu
Joanna M. Gonzalez	Miami	jmg442@miami.edu
John A. Phillips III	Vanderbilt	John.a.phillips@vumc.org
John Carey	University of Utah	john.carey@hsc.utah.edu
John J. Mulvihill	NIH UDP	johmulvihill@gmail.com

Joie Davis	NIH UDP, NHGRI	jdavis@niaid.nih.gov
Jonathan A. Bernstein	Stanford	Jon.Bernstein@stanford.edu
Jordan Whitlock	UAB DMCC	jbarham3@uab.edu
Jose Abdenur	UCI/CHOC	JAbdenur@choc.org
Joseph Loscalzo	Harvard	jloscalzo@partners.org
Joy D. Cogan	Vanderbilt	joy.cogan@vumc.org
Julian A. Martínez-Agosto	UCLA	julianmartinez@mednet.ucla.edu
Justin Alvey	University of Utah	justin.alvey@hsc.utah.edu
Kahlen Darr	Mayo Clinic	Darr.Kahlen@mayo.edu
Kaitlin Callaway	UAB	kcallaway@uabmc.edu
Kathleen A. Leppig	PNW	leppig@uw.edu
Kathleen Sullivan	CHOP	sullivank@chop.edu
Kathy Sisco	WUSTL Clinical	siscok@wustl.edu
Kathyrn Singh	UCI/CHOC	kesingh@hs.uci.edu
Katrina Dipple	PNW	katrina.dipple@seattlechildrens.org
Kayla M. Treat	IU	ktreat@iuhealth.org
Kelly Hassey	CHOP	hasseyk@chop.edu
Kelly Schoch	Duke	kelly.schoch@duke.edu
Kevin S. Smith	Stanford	kssmith@stanford.edu

Khurram Liaqat	IU	kliaqat@iu.edu
Kim Worley	BCM Clinical	kworley@bcm.edu
Kimberly Ezell	Vanderbilt	kimberly.ezell@vumc.org
Kimberly LeBlanc	Harvard DMCC	kimberly_leblanc@hms.harvard.edu
Kumarie Latchman	Miami	kxl604@med.miami.edu
Lance H. Rodan	Harvard	lance.rodan@childrens.harvard.edu
Laura Pace	University of Utah	laura.pace@hsc.utah.edu
Laurel A. Cobban	Harvard	lcobban@bwh.harvard.edu
Lauren C. Briere	Harvard	lbriere@partners.org
Leoyklang Petcharet	NIH UDP, NHGRI	petcharat.leoyklang@nih.gov
LéShon Peart	Miami	L.peart@med.miami.edu
Lili Mantcheva	IU	lmantche@iu.edu
Lilianna Solnica-Krezel	WUSTL MOSC	solnical@wustl.edu
Lindsay C. Burrage	BCM Clinical	burrage@bcm.edu
Lindsay Mulvihill	Mayo Clinic	mulvihill.lindsay@mayo.edu
Lisa Schimmenti	Mayo Clinic	Schimmenti.Lisa@mayo.edu
Lisa T. Emrick	BCM Clinical	emrick@bcm.edu
Lorenzo Botto	University of Utah	lorenzo.botto@hsc.utah.edu
Lorraine Potocki	BCM Clinical	lpotocki@bcm.edu

Lynette Rives	Vanderbilt	lynette.c.rives@vumc.org
Lynne A. Wolfe	NIH UDP, NHGRI	lynne.wolfe@nih.gov
Manish J. Butte	UCLA	mbutte@mednet.ucla.edu
Margaret Delgado	NIH UDP, NHGRI	margaret.delgado@nih.gov
Maria T. Acosta	NIH UDP	acostam@nhgri.nih.gov
Marie Morimoto	NIH UDP, NHGRI	marie.morimoto@nih.gov
Mariko Nakano-Okuno	UAB DMCC	marikonk@uab.edu
Mark Wener	PNW	wener@uw.edu
Marla Sabaii	NIH UDP, NHGRI	marla.sabaii@nih.gov
Martha Horike-Pyne	PNW	mpyne@medicine.washington.edu
Martin G. Martin	UCLA	mmartin@mednet.ucla.edu
Martin Rodriguez	UAB	rodriguez@uabmc.edu
Matthew Coggins	Harvard	mcoggins@bwh.harvard.edu
Matthew Might	UAB DMCC	might@uab.edu
Matthew T. Wheeler	Stanford	wheelerm@stanford.edu
Maura Ruzhnikov	Stanford	ruzhnikov@stanford.edu
MayChristine V. Malicdan	NIH UDP, NHGRI	maychristine.malicdan@nih.gov
Meghan C. Halley	Stanford	mhalley@stanford.edu
Melissa Walker	Harvard	walker.melissa@mgh.harvard.edu

Michael Bamshad	PNW	mbamshad@uw.edu
Michael F. Wangler	BCM MOSC	mw147467@bcm.edu
Miguel Almalvez	UCI/CHOC	malmalve@hs.uci.edu
Mohamad Mikati	Duke	mohamad.mikati@duke.edu
Monika Weisz Hubshman	BCM Clinical	hubshman@bcm.edu
Monte Westerfield	UO MOSC	monte@uoneuro.uoregon.edu
Mustafa Tekin	Miami	mtekin@miami.edu
Naghmeh Dorrani	UCLA	ndorrani@mednet.ucla.edu
Neil H. Parker	UCLA	nhparker@mednet.ucla.edu
Neil Hanchard	NIH UDP, NHGRI	neil.hanchard@nih.gov
Nicholas Borja	Miami	nborja@med.miami.edu
Nicola Longo	University of Utah	nicola.longo@hsc.utah.edu
Nicole M. Walley	Duke	nicole.walley@duke.edu
Nina Movsesyan	UCI/CHOC	nmovsesyan@choc.org
Nitsuh K. Dargie	PNW	nitsuhk@medicine.washington.edu
Oguz Kanca	BCM MOSC	Oguz.Kanca@bcm.edu
Orpa Jean-Marie	NIH UDP, NHGRI	orpa.jean-marie@nih.gov
Page C. Goddard	Stanford	pgoddard@stanford.edu
Paolo Moretti	University of Utah	paolo.moretti@hsc.utah.edu

Patricia A. Ward	BCM Sequencing	pward@bcm.edu
Patricia Dickson	WUSTL Clinical	pdickson@wustl.edu
Paul G. Fisher	Stanford	pfisher@stanford.edu
Pengfei Liu	BCM Sequencing	pliu@baylorgenetics.com
Peter Byers	PNW	pbyers@uw.edu
Pinar Bayrak-Toydemir	University of Utah/ARUP	pinar.bayrak-toydemir@arup.com
Precilla D'Souza	NIH UDP	precilla.d'souza@nih.gov
Queenie Tan	Mayo Clinic	Tan.KhoonGheeQueenie@mayo.edu
Rachel A. Ungar	Stanford	raungar@stanford.edu
Rachel Mahoney	Harvard DMCC	rachel_mahoney@hms.harvard.edu
Ramakrishnan Rajagopalan	CHOP	rajagopalanr@chop.edu
Raquel L. Alvarez	Stanford	raquela1@stanford.edu
Rebecca C. Spillmann	Duke	rebecca.crimian@duke.edu
Rebecca Ganetzky	CHOP	ganetzkyr@chop.edu
Rebecca Overbury	University of Utah	rebecca.overbury@hsc.utah.edu
Rebekah Barrick	UCI/CHOC	rebekah.barrick@choc.org
Richard A. Lewis	BCM Clinical	rlewis@bcm.edu
Richard L. Maas	Harvard	maas@genetics.med.harvard.edu
Rizwan Hamid	Vanderbilt	rizwan.hamid@vumc.org

Rong Mao	University of Utah/ARUP	rong.mao@aruplab.com
Ronit Marom	BCM Clinical	ronit.marom@bcm.edu
Rosario I. Corona	UCLA	rcoronadelafuente@mednet.ucla.edu
Russell Butterfield	University of Utah	russell.butterfield@hsc.utah.edu
Sam Sheppeard	PNW	samshep@uw.edu
Sanaz Attaripour	UCI/CHOC	sattarip@hs.uci.edu
Seema R. Lalani	BCM Clinical	seemal@bcm.edu
Serena Neumann	Vanderbilt	serena.neumann@vumc.org
Shamika Ketkar	BCM Clinical	ketkar@bcm.edu
Shamil R. Sunyaev	Harvard DMCC	ssunyaev@hms.harvard.edu
Shilpa N. Kobren	Harvard DMCC	Shilpa_Kobren@hms.harvard.edu
Shinya Yamamoto	BCM MOSC	yamamoto@bcm.edu
Shirley Sutton	Stanford	scsutton@stanford.edu
Shruti Marwaha	Stanford	mshruti@stanford.edu
Sirisak Chanprasert	PNW	sirisc@uw.edu
Stanley F. Nelson	UCLA	snelson@ucla.edu
Stephan Zuchner	Miami	szuchner@miami.edu
Stephanie Bivona	Miami	sab355@miami.edu
Stephanie M. Ware	IU	stware@iu.edu

Stephen Pak	WUSTL MOSC	stephen.pak@email.wustl.edu
Steven Boyden	University of Utah	steven.boyden@genetics.utah.edu
Suman Jayadev	PNW	sumie@uw.edu
Surendra Dasari	Mayo Clinic	dasari.surendra@mayo.edu
Susan Korrick	Harvard	skorrick@bwh.harvard.edu
Suzanne Sandmeyer	UCI/CHOC	sbsandme@hs.uci.edu
Tahseen Mozaffar	UCI/CHOC	mozaffar@hs.uci.edu
Tammi Skelton	UAB	tlskelton@uabmc.edu
Tara Wenger	PNW	tara.wenger@seattlechildrens.org
Terra R. Coakley	Stanford	tcoakley@stanford.edu
Thomas Cassini	Vanderbilt	thomas.a.cassini@vumc.org
Thomas J. Nicholas	University of Utah	thomas.nicholas@utah.edu
Timothy Schedl	WUSTL MOSC	ts@wustl.edu
Tiphanie P. Vogel	BCM Clinical	tiphanie.vogel@bcm.edu
Vaidehi Jobanputra	Columbia	vj2004@cumc.columbia.edu
Valerie V. Maduro	NIH UDP	vbraden@mail.nih.gov
Vandana Shashi	Duke	vandana.shashi@duke.edu
Virginia Sybert	PNW	flk01@uw.edu
Vishnu Cuddapah	CHOP	cuddapahv@chop.edu

Wendy Introne	NIH UDP, NHGRI	wintrone@nhgri.nih.gov
Wendy Raskind	PNW	wendyrun@uw.edu
Willa Thorson	Miami	wthorson@miami.edu
William A. Gahl	NIH UDP, NHGRI	gahlw@mail.nih.gov
William E. Byrd	UAB DMCC	webyrd@gmail.com
William J. Craigen	BCM Clinical	wcraigen@bcm.edu
Yan Huang	NIH UDP, NHGRI	yan.huang@nih.gov
Yigit Karasozen	UCLA	Ykarasozen@mednet.ucla.edu

Supplementary References

1. Noriega MA, S. A. *Trisomy 13*. (StatPearls Publishing, Updated 2023 Aug 13).
2. Tsutsumi, M. *et al.* A female patient with retinoblastoma and severe intellectual disability carrying an X;13 balanced translocation without rearrangement in the RB1 gene: a case report. *BMC Med Genomics* **12**, 182, doi:10.1186/s12920-019-0640-2 (2019).
3. Mefford, H. C. *et al.* Rare copy number variants are an important cause of epileptic encephalopathies. *Ann Neurol* **70**, 974-985, doi:10.1002/ana.22645 (2011).
4. Winnepenninckx, B. *et al.* CGG-repeat expansion in the DIP2B gene is associated with the fragile site FRA12A on chromosome 12q13.1. *Am J Hum Genet* **80**, 221-231, doi:10.1086/510800 (2007).
5. Singh, R., Scheffer, I. E., Crossland, K. & Berkovic, S. F. Generalized epilepsy with febrile seizures plus: a common childhood-onset genetic epilepsy syndrome. *Ann Neurol* **45**, 75-81, doi:10.1002/1531-8249(199901)45:1<75::aid-art13>3.0.co;2-w (1999).
6. Heron, S. E. *et al.* Genetic variation of CACNA1H in idiopathic generalized epilepsy. *Ann Neurol* **55**, 595-596, doi:10.1002/ana.20028 (2004).
7. Heron, S. E. *et al.* Extended spectrum of idiopathic generalized epilepsies associated with CACNA1H functional variants. *Ann Neurol* **62**, 560-568, doi:10.1002/ana.21169 (2007).
8. Morel Swols D, T. M. KBG Syndrome. *GeneReviews* (1993-2023).
9. Roston A, G. W. SETD1B-Related Neurodevelopmental Disorder. *GeneReviews* (1993-2023).
10. JA., F. TET3-Related Beck-Fahrner Syndrome. . *GeneReviews* (1993-2023).
11. Stevenson, R. E. *et al.* UBE2A-related X-linked intellectual disability. *Clin Dysmorphol* **28**, 1-6, doi:10.1097/MCD.0000000000000242 (2019).
12. Schwartz CE, P. A., Kutler MJ. Snyder-Robinson Syndrome. *GeneReviews®* (2020).

13 Parkinson Progression Marker, I. The Parkinson Progression Marker Initiative (PPMI). *Prog Neurobiol* **95**, 629-635, doi:10.1016/j.pneurobio.2011.09.005 (2011).

14 Dong, Q. *et al.* Genome-wide association studies identify novel genetic loci for epigenetic age acceleration among survivors of childhood cancer. *Genome Med* **14**, 32, doi:10.1186/s13073-022-01038-6 (2022).

15 Vaisvila, R. *et al.* Enzymatic methyl sequencing detects DNA methylation at single-base resolution from picograms of DNA. *Genome Res* **31**, 1280-1289, doi:10.1101/gr.266551.120 (2021).

16 Danecek, P. *et al.* Twelve years of SAMtools and BCFtools. *Gigascience* **10**, doi:10.1093/gigascience/giab008 (2021).

17 MethylDackel (https://github.com/dpryan79/MethylDackel).

18 Ryan, P. *et al.* Scaling accurate genetic variant discovery to tens of thousands of samples. *bioRxiv*, 201178, doi:10.1101/201178 (2018).

19 Karczewski, K. J. *et al.* The mutational constraint spectrum quantified from variation in 141,456 humans. *Nature* **581**, 434-443, doi:10.1038/s41586-020-2308-7 (2020).

20 Karczewski, K. J. *et al.* The ExAC browser: displaying reference data information from over 60 000 exomes. *Nucleic Acids Res* **45**, D840-D845, doi:10.1093/nar/gkw971 (2017).

21 Fu, W. *et al.* Analysis of 6,515 exomes reveals the recent origin of most human protein-coding variants. *Nature* **493**, 216-220, doi:10.1038/nature11690 (2013).

22 Genomes Project, C. *et al.* A global reference for human genetic variation. *Nature* **526**, 68-74, doi:10.1038/nature15393 (2015).

23 Liu, X., Jian, X. & Boerwinkle, E. dbNSFP: a lightweight database of human nonsynonymous SNPs and their functional predictions. *Hum Mutat* **32**, 894-899, doi:10.1002/humu.21517 (2011).

24 Landrum, M. J. *et al.* ClinVar: improving access to variant interpretations and supporting evidence. *Nucleic Acids Res* **46**, D1062-D1067, doi:10.1093/nar/gkx1153 (2018).

25 Li, Q. & Wang, K. InterVar: Clinical Interpretation of Genetic Variants by the 2015 ACMG-AMP Guidelines. *Am J Hum Genet* **100**, 267-280, doi:10.1016/j.ajhg.2017.01.004 (2017).

26 Wang, K., Li, M. & Hakonarson, H. ANNOVAR: functional annotation of genetic variants from high-throughput sequencing data. *Nucleic Acids Res* **38**, e164, doi:10.1093/nar/gkq603 (2010).

27 Jaganathan, K. *et al.* Predicting Splicing from Primary Sequence with Deep Learning. *Cell* **176**, 535-548 e524, doi:10.1016/j.cell.2018.12.015 (2019).

28 McLaren, W. *et al.* The Ensembl Variant Effect Predictor. *Genome Biol* **17**, 122, doi:10.1186/s13059-016-0974-4 (2016).

29 Ewels, P. A. *et al.* The nf-core framework for community-curated bioinformatics pipelines. *Nat Biotechnol* **38**, 276-278, doi:10.1038/s41587-020-0439-x (2020).

30 Giacopuzzi, E., Popitsch, N. & Taylor, J. C. GREEN-DB: a framework for the annotation and prioritization of non-coding regulatory variants from whole-genome sequencing data. *Nucleic Acids Res* **50**, 2522-2535, doi:10.1093/nar/gkac130 (2022).

31 Yépez, V. A., Murdock, D. R. & Lee, B. Gene expression counts from fibroblast, strand-specific, BCM UDN. *Zenodo*, doi:10.5281/zenodo.3963474 (2020).

32 Levy, M. A. *et al.* Functional correlation of genome-wide DNA methylation profiles in genetic neurodevelopmental disorders. *Hum Mutat* **43**, 1609-1628, doi:10.1002/humu.24446 (2022).

33 Levy, M. A. *et al.* Novel diagnostic DNA methylation episignatures expand and refine the epigenetic landscapes of Mendelian disorders. *Human Genetics and Genomics Advances*, doi:10.1016/j.xhgg.2021.100075 (2021).
