# Supplementary material for: Diagnostic utility of DNA methylation analysis in genetically unsolved pediatric epilepsies and CHD2 episignature refinement
Source: Nat Commun. 2024 Aug 6;15:6524. doi: 10.1038/s41467-024-50159-6 (PMC11303402; doi:10.1038/s41467-024-50159-6)
Supplement: Supplementary file 3 — Description of Additional Supplementary Files [file 41467_2024_50159_MOESM3_ESM.pdf]

**Description of Additional Supplementary Files**

File Name: Supplementary Data 1
Description: Cohort details, methylation array quality control and filtering numbers.

File Name: Supplementary Data 2
Description: Detailed variant table for rare DMRs and episignatures.

File Name: Supplementary Data 3
Description: List of all rare outlier DMRs for the autosomes and chrX.

File Name: Supplementary Data 4
Description: Comparison of CpG coverage for DMRs validated by targeted EM-seq.

File Name: Supplementary Data 5
Description: Variant table from trio sequencing analysis for the individual with a X;13 translocation.

File Name: Supplementary Data 6
Description: X-inactivation assay for mother with BCLAF3 hypermethylation.

File Name: Supplementary Data 7
Description: Probe information for CHD2 450K and 850K episignatures.

File Name: Supplementary Data 8
Description: CHD2 DMR master list.

File Name: Supplementary Data 9
Description: List of randomly generated genomic coordinates for comparison of functional annotations with CHD2 DMRs.

File Name: Supplementary Data 10
Description: Enrichment values of CHD2 episignature probes and DMRs.
