# Supplementary material for: Diagnostic utility of DNA methylation analysis in genetically unsolved pediatric epilepsies and CHD2 episignature refinement
Source: Nat Commun. 2024 Aug 6;15:6524. doi: 10.1038/s41467-024-50159-6 (PMC11303402; doi:10.1038/s41467-024-50159-6)
Supplement: Supplementary file 14 — Reporting Summary [file 41467_2024_50159_MOESM14_ESM.pdf]

nature portfolio

Corresponding author(s): Heather Mefford, Bekim Sadikovic

Last updated by author(s): May 22, 2024

Reporting Summary

Nature Portfolio wishes to improve the reproducibility of the work that we publish. This form provides structure for consistency and transparency in reporting. For further information on Nature Portfolio policies, see our Editorial Policies and the Editorial Policy Checklist.

Statistics

For all statistical analyses, confirm that the following items are present in the figure legend, table legend, main text, or Methods section.

n/a	Confirmed	
☐	☒	The exact sample size (*n*) for each experimental group/condition, given as a discrete number and unit of measurement
☐	☒	A statement on whether measurements were taken from distinct samples or whether the same sample was measured repeatedly
☐	☒	The statistical test(s) used AND whether they are one- or two-sided<br>*Only common tests should be described solely by name; describe more complex techniques in the Methods section.*
☐	☒	A description of all covariates tested
☒	☐	A description of any assumptions or corrections, such as tests of normality and adjustment for multiple comparisons
☐	☒	A full description of the statistical parameters including central tendency (e.g. means) or other basic estimates (e.g. regression coefficient) AND variation (e.g. standard deviation) or associated estimates of uncertainty (e.g. confidence intervals)
☐	☒	For null hypothesis testing, the test statistic (e.g. *F*, *t*, *r*) with confidence intervals, effect sizes, degrees of freedom and *P* value noted<br>*Give P values as exact values whenever suitable.*
☒	☐	For Bayesian analysis, information on the choice of priors and Markov chain Monte Carlo settings
☒	☐	For hierarchical and complex designs, identification of the appropriate level for tests and full reporting of outcomes
☐	☒	Estimates of effect sizes (e.g. Cohen's *d*, Pearson's *r*), indicating how they were calculated

Our web collection on statistics for biologists contains articles on many of the points above.

Software and code

Policy information about availability of computer code

Data collection	N/A
Data analysis	MethylMiner contains code for QC, processing, and data analysis of rare DMRs from raw .idat methylation array data and is available for use on github (https://github.com/stjude-biohackathon/MethylMiner). Mefford lab github repository contains scripts relevant for analyses as described (https://github.com/MeffordLab/2024_GenomeWideMethylationPaper).

For manuscripts utilizing custom algorithms or software that are central to the research but not yet described in published literature, software must be made available to editors and reviewers. We strongly encourage code deposition in a community repository (e.g. GitHub). See the Nature Portfolio guidelines for submitting code & software for further information.

Data

Policy information about availability of data

All manuscripts must include a data availability statement. This statement should provide the following information, where applicable:

- Accession codes, unique identifiers, or web links for publicly available datasets
- A description of any restrictions on data availability
- For clinical datasets or third party data, please ensure that the statement adheres to our policy

Raw .idat methylation array data will be available on dbGaP for individuals with unsolved DEE who have consented to controlled data access (n=365) for study of epilepsy and candidate genes.

Research involving human participants, their data, or biological material

Policy information about studies with human participants or human data. See also policy information about sex, gender (identity/presentation), and sexual orientation and race, ethnicity and racism.

Reporting on sex and gender	Sex was reported and calculated from methylation array data (based off intensities of X and Y probes) for the purposes of the analysis (males and females separated to analyze the sex chromosomes).
Reporting on race, ethnicity, or other socially relevant groupings	patients were recruited for clinical phenotype of DEE and were not excluded (or included) on the basis of race, ethnicity, sex, or other socially relevant groupings
Population characteristics	All patients have early-onset epilepsy and developmental delays, consistent with a DEE diagnosis
Recruitment	Affected individuals were recruited or referred to the study by clinicians on the basis of having a clinical presentation consistent with developmental and epileptic enceaphalopathy with onset in childhood.
Ethics oversight	This study was approved by IRBs at St. Jude Children's Research Hospital, Austin Health (Australia), the University of Washington (UW), and the National Institutes of Health (NIH).

Note that full information on the approval of the study protocol must also be provided in the manuscript.

Field-specific reporting

Please select the one below that is the best fit for your research. If you are not sure, read the appropriate sections before making your selection.

☒ Life sciences ☐ Behavioural & social sciences ☐ Ecological, evolutionary & environmental sciences

For a reference copy of the document with all sections, see nature.com/documents/nr-reporting-summary-flat.pdf

Life sciences study design

All studies must disclose on these points even when the disclosure is negative.

Sample size	n=582 individuals with unsolved DEE
Data exclusions	Exclusions were based on data quality (PCA outlier, cell type composition, >100 outlier DMRs on autosomes from initial analysis). The exact samples excluded are detailed in Table S1B.
Replication	Replicate array data was obtained for n=29 individuals. Details about the concordance of rare DMRs across these individuals is located in the Supplemental Materials and Methods. Additionally, the diagnostic yield was validated on an independent cohort of n=66 individuals during the revision process (and is now a part of the main cohort).
Randomization	N/A
Blinding	N/A

Reporting for specific materials, systems and methods

We require information from authors about some types of materials, experimental systems and methods used in many studies. Here, indicate whether each material, system or method listed is relevant to your study. If you are not sure if a list item applies to your research, read the appropriate section before selecting a response.

Materials & experimental systems

n/a	Involved in the study	
☒	☐	Antibodies
☒	☐	Eukaryotic cell lines
☒	☐	Palaeontology and archaeology
☒	☐	Animals and other organisms
☒	☐	Clinical data
☒	☐	Dual use research of concern
☒	☐	Plants

Methods

n/a	Involved in the study	
☒	☐	ChIP-seq
☒	☐	Flow cytometry
☒	☐	MRI-based neuroimaging

Plants

Seed stocks	*Report on the source of all seed stocks or other plant material used. If applicable, state the seed stock centre and catalogue number. If plant specimens were collected from the field, describe the collection location, date and sampling procedures.*
Novel plant genotypes	*Describe the methods by which all novel plant genotypes were produced. This includes those generated by transgenic approaches, gene editing, chemical/radiation-based mutagenesis and hybridization. For transgenic lines, describe the transformation method, the number of independent lines analyzed and the generation upon which experiments were performed. For gene-edited lines, describe the editor used, the endogenous sequence targeted for editing, the targeting guide RNA sequence (if applicable) and how the editor was applied.*
Authentication	*Describe any authentication procedures for each seed stock used or novel genotype generated. Describe any experiments used to assess the effect of a mutation and, where applicable, how potential secondary effects (e.g. second site T-DNA insertions, mosiacism, off-target gene editing) were examined.*
